# MEK activation modulates glycolysis and supports suppressive myeloid cells in TNBC

**DOI:** 10.1172/jci.insight.134290

**Published:** 2020-08-06

**Authors:** Derek A. Franklin, Joe T. Sharick, Paula I. Ericsson-Gonzalez, Violeta Sanchez, Phillip T. Dean, Susan R. Opalenik, Stefano Cairo, Jean-Gabriel Judde, Michael T. Lewis, Jenny C. Chang, Melinda E. Sanders, Rebecca S. Cook, Melissa C. Skala, Jennifer Bordeaux, Jehovana Orozco Bender, Christine Vaupel, Gary Geiss, Douglas Hinerfeld, Justin M. Balko

**Affiliations:** 1 Department of Medicine, Vanderbilt University Medical Center, Nashville, Tennessee, USA.; 2 Department of Biomedical Engineering, School of Engineering, Vanderbilt University, Nashville, Tennessee, USA.; 3 Morgridge Institute for Research, University of Wisconsin–Madison, Wisconsin, USA.; 4 Breast Cancer Research Program, Vanderbilt-Ingram Cancer Center, Nashville, Tennessee, USA.; 5 Xentech, Genopole Campus 3, Evry, France.; 6 Baylor College of Medicine, Houston, Texas, USA.; 7 Department of Radiation Oncology, Houston Methodist Research Institute, Houston, Texas, USA.; 8 Department of Pathology, Microbiology and Immunology and; 9 Department of Cell and Developmental Biology, Vanderbilt University Medical Center, Nashville Tennessee, USA.; 10 Department of Biomedical Engineering, College of Engineering, University of Wisconsin–Madison, Wisconsin, USA.; 11 Navigate BioPharma Services, Inc., Carlsbad, California, USA.; 12 NanoString Technologies, Seattle, Washington, USA.

**Keywords:** Immunology, Oncology, Breast cancer

## Abstract

Triple-negative breast cancers (TNBCs) are heterogeneous and aggressive, with high mortality rates. TNBCs frequently respond to chemotherapy, yet many patients develop chemoresistance. The molecular basis and roles for tumor cell–stromal crosstalk in establishing chemoresistance are complex and largely unclear. Here we report molecular studies of paired TNBC patient–derived xenografts (PDXs) established before and after the development of chemoresistance. Interestingly, the chemoresistant model acquired a distinct *KRAS^Q61R^* mutation that activates K-Ras. The chemoresistant *KRAS*-mutant model showed gene expression and proteomic changes indicative of altered tumor cell metabolism. Specifically, *KRAS*-mutant PDXs exhibited increased redox ratios and decreased activation of AMPK, a protein involved in responding to metabolic homeostasis. Additionally, the chemoresistant model exhibited increased immunosuppression, including expression of CXCL1 and CXCL2, cytokines responsible for recruiting immunosuppressive leukocytes to tumors. Notably, chemoresistant *KRAS-*mutant tumors harbored increased numbers of granulocytic myeloid-derived suppressor cells (gMDSCs). Interestingly, previously established Ras/MAPK-associated gene expression signatures correlated with myeloid/neutrophil-recruiting CXCL1/2 expression and negatively with T cell–recruiting chemokines (CXCL9/10/11) across patients with TNBC, even in the absence of KRAS mutations. MEK inhibition induced tumor suppression in mice while reversing metabolic and immunosuppressive phenotypes, including chemokine production and gMDSC tumor recruitment in the chemoresistant KRAS-mutant tumors. These results suggest that Ras/MAPK pathway inhibitors may be effective in some breast cancer patients to reverse Ras/MAPK-driven tumor metabolism and immunosuppression, particularly in the setting of chemoresistance.

## Abstract 2

Longitudinally-derived TNBC xenografts identify acquired KRAS mutation/MEK activation as a driving feature of CXCL1/2-mediated MDSC recruitment.

## Graphical abstract

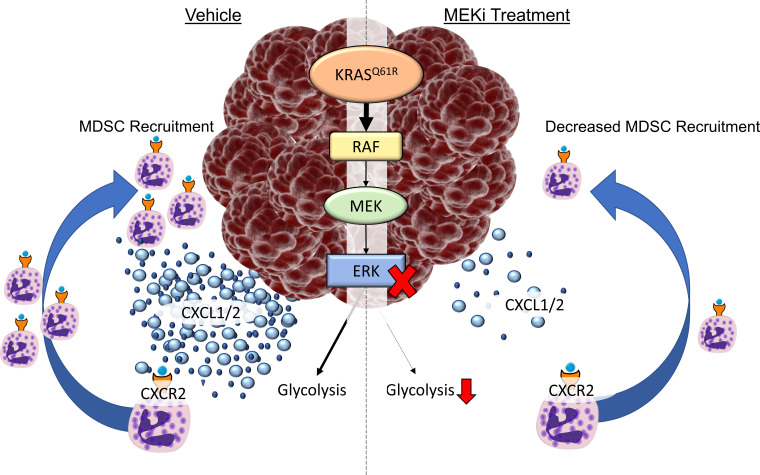

## Introduction

Triple-negative breast cancer (TNBC) is a unique clinical subtype of breast cancer characterized by uniform lack of estrogen receptor and progesterone receptor expression and the absence of human epidermal growth factor receptor amplification. TNBC is characterized by diverse chromosomal aberrations and frequent loss of tumor suppressor p53 functions but infrequent druggable mutations. This genomic instability contributes to its heterogeneity and the perception that TNBC is a diverse collection of malignancies rather than a unique molecular entity (1). TNBC also demonstrates high rates of mortality, primarily due to its propensity to metastasize to visceral organs early in the clinical course.

Consistent with the lack of recurrently altered targets at the genomic level, there is a paucity of approved molecularly targeted agents for TNBC. As such, TNBC is primarily treated with chemotherapeutics. Nonetheless, several distinct molecular pathways have been implicated in TNBC phenotypes. Of these, the signaling cascade through the Ras-Raf-Mek-Erk (Ras/MAPK) pathway is known to be oncogenic and involved in the development of multiple other cancers (2–4). Ras/MAPK signaling can be activated by a variety of mechanisms, including activating mutations in *KRAS*, *NRAS*, *HRAS*, or *BRAF*, which are commonly mutated in cancers but are rarely observed in primary breast cancers (5). Indeed, the Ras/MAPK pathway has been shown to be primarily activated in breast cancer via loss of negative regulation (6, 7). However, the abundance of Ras/MAPK mutations/alterations in metastatic breast cancer patients may be increased compared with primary breast cancers but is less well studied (8). Ras/MAPK signaling contributes to cancer stem cell–like phenotypes (7, 9), immune evasion (10, 11), metabolic alterations (12), and progression/metastasis (13). Thus, the development of targeted Ras/MAPK therapeutics in TNBC requires additional study on which tumors are likely to respond, what is the most effective way to incorporate Ras/MAPK inhibitors (i.e., MEK inhibitors; MEKi) into therapy, and which molecular phenotypes can be effectively targeted.

To address these questions, we used a panel of TNBC patient–derived xenograft (PDX) models to evaluate responsiveness to combined treatment with MEKi and taxane-based chemotherapy. Taxanes are commonly used for treatment of primary and metastatic TNBC. The Ras/MAPK pathway has been implicated in chemotherapeutic resistance (7, 14–16), and synergy between MEKi and taxanes has been reported in the literature (6, 17). Furthermore, ongoing clinical trials are exploring combinations of taxanes and MEKi, with promising results (18). Although Ras/MAPK activating mutations are rare in breast cancer (5), they can occur infrequently (19), and we and others have previously reported loss of Ras/MAPK negative regulators (*DUSP4*, *NF1*, *RND1*) in breast cancer as a mechanism of pathway activation (6, 7, 20, 21). Across 4 assayed models, only 1 demonstrated a high degree of responsiveness to MEKi, and this model was part of a matched pair of PDXs derived from the same patient, before and after development of resistance to chemotherapy (22). Exploring the chemoresistant model and its parental PDX in further detail, we identified distinct gene expression and proteomic changes driven by Ras/MAPK signaling to promote tumor growth and immune evasion, specifically though recruitment of myeloid-derived suppressor cells (MDSCs), via a CXCR2-dependent process. Although our data were generated in an immunocompromised mouse model, we feel that the identification of novel mechanisms by which MAPK signaling alters the tumor immune microenvironment will be invaluable to further therapeutic development in TNBC.

## Results

### Sensitivity of TNBC PDX models to doxorubicin/cyclophosphamide → taxane ± MEK inhibition.

**Figure 1:**
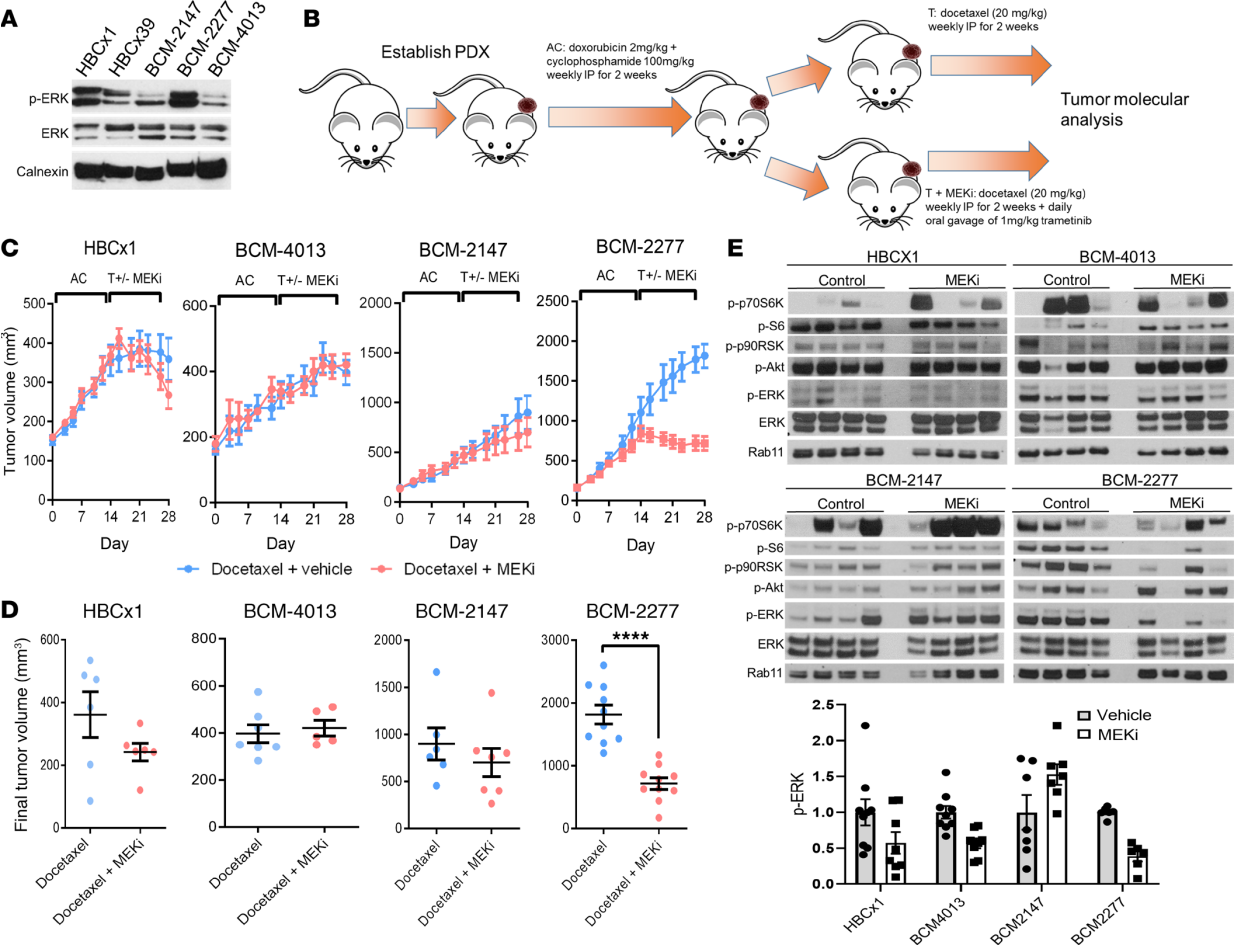
Therapeutic and transcriptional response of TNBC PDX models to standard chemotherapy or standard chemotherapy with MEK inhibition. (**A**) Representative Western blot of untreated tumors from PDX models. (**B**) Schematic for treatment of PDX models. (**C**) Tumor growth curves for PDX models. (*n* = 5–10 per condition.) AC, adriamycin (doxorubicin) and cyclophosphamide; T, taxane (docetaxel). (**D**) Final tumor volumes at 28 days. (*n* = 5–10 per condition.) *P* value represents 2-sample 2-tailed *t* test. (**E**) Western blot of representative treated tumors from **C** and **D**. ****P < 0.0001.

We sought to determine whether addition of a MEKi improved responses to a standard chemotherapy combination regimen in the setting of chemoresistance and to further explore the effects of MEKi treatment on the mammary tumor microenvironment. We began by exploring 5 TNBC PDX models by Western blot analysis to determine the activation status of the Ras/MAPK pathway (Figure 1A). PDX models BCM-2277 and HBCx1 exhibited the highest activation of phosphorylated ERK1/2 (p-ERK) and thus were selected for further analysis, along with BCM-4013 and BCM-2147 as controls. Of note, BCM-2147 and BCM-2277 were derived from the same patient. Specifically, BCM-2147 was isolated before chemotherapy treatment and progression, whereas BCM-2277 was derived after progression.

Using an accelerated therapeutic regimen mirroring an approved therapy for TNBC (doxorubicin/cyclophosphamide **→** taxane; AC**→**T) with or without the addition of the MEKi trametinib in the taxane (docetaxel) phase (AC**→**TM), we tracked tumor growth over time. After 4 weeks of therapy (2 weeks of weekly doxorubicin and cyclophosphamide and 2 weeks of docetaxel ± trametinib), tumors were harvested for molecular analysis (Figure 1B). Interestingly, the only model that exhibited statistically significant reductions in tumor growth with MEKi over the 2-week treatment period were the postchemotherapy/progression BCM-2277 tumors (*P* < 0.0001) (Figure 1, C and D). HBCx1, which also showed MEK activation by Western blot, and BCM-2147 showed marginal effects of MEKi that were not statistically significant during the treatment period. HBCx1 tumors also appeared to be more sensitive to taxane treatment than the other models tested. Western blot analysis of treated tumor replicates demonstrated that MEKi treatment decreased both p-ERK and p-S6 ribosomal protein, a marker of increased translation and a downstream target of MAPK signaling, only in BCM-2277, whereas diminished p-ERK but not p-S6 was observed in HBCx1 (Figure 1E). Previous studies have shown that MEKi treatment activates prosurvival feedback loops in various contexts, which may partially explain the lack of MEKi-specific responses within HBCx1 tumors (23, 24). As mentioned previously, HBCx1 tumors appear to be sensitive to taxane therapy, which could also make observing MEKi-specific responses more difficult.

### Molecular metabolic imaging of tumor organoids reveals a link between MEK activation and glycolysis.

To molecularly characterize the residual tumors from PDX models treated with AC**→**T or AC**→**TM, we collected tumor samples at the end of the 4-week treatment. We then used NanoString 3D Biology technology to simultaneously analyze common DNA alterations (104 single nucleotide variants across 25 genes), RNA expression (192 cancer-targeted mRNAs), and protein expression/activation (26 proteins and phosphoproteins) from a single formalin-fixed, paraffin-embedded (FFPE) sample (Supplemental Figure 1; supplemental material available online with this article; https://doi.org/10.1172/jci.insight.134290DS1). Replicate samples from the same tumor demonstrated high reproducibility (*r* > 0.99) with lower correlations observed between tumors from the same model and even lower correlations between tumors from different models, as expected (Supplemental Figure 2). We initially focused on analyzing the gene expression data generated from the 3D Biology analysis by performing unbiased clustering across the 192 transcripts analyzed. Importantly, each model clustered separately, suggesting gene expression differences between each PDX, as expected (Supplemental Figure 3A). However, only BCM-2277 clustered by MEKi treatment, suggesting that MEKi treatment did not affect the other PDXs as uniformly. HBCx1 did exhibit a partial response to MEKi treatment but had a single MEKi-treated tumor that clustered with the untreated tumor samples. These data suggest that (a) the models are transcriptionally distinct from one another and (b) a substantial effect of MEKi was observed only in BCM-2277, and to a lesser extent in HBCx1, replicating our tumor growth and protein expression data. A correlation matrix across samples also demonstrated that transcription in BCM-2277 bore general resemblance to its parental model, BCM-2147. Interestingly, MEKi treatment in BCM-2277 resulted in gene expression profiles that exhibited increased similarity with the parental BCM-2147 model (Supplemental Figure 3A). These data suggest that MEK activation was a driving force in the molecular differences observed between the 2 models in response to combination chemotherapy treatment and supported an association of Ras/MAPK activation with chemotherapeutic resistance.

DNA mutation analysis was completed by using PCR to amplify genomic loci within 25 genes frequently altered in solid tumors prior to hybridization with specially designed probes for detection of short nucleotide variants. This analysis did not identify any alterations in BCM-2147, HBCx1, or BCM-4013 samples. Yet, a single *KRAS^Q61R^* mutation was present in 8/8 BCM-2277 model samples (Supplemental Figure 3B). This mutation is known to be oncogenic (25) but is generally rare in primary human tumors (Supplemental Figure 4) (26). Interestingly, a recent report identified *KRAS* codon 61 mutations as arising specifically under therapeutic selection, rather than as direct tumor initiators (27). Analysis of thousands of genetic profiles of human breast cancers revealed an enrichment of *KRAS* mutations in metastatic breast cancers versus primary breast cancer; however, these mutations were all codon 12 and codon 13 mutations (Supplemental Figure 4). Yet, limited data exist on chemotherapy-resistant breast cancers profiled at the end of therapy, representing a knowledge gap of KRAS mutational rates in that population. Thus, the prevalence of *KRAS* codon 61 mutations in breast cancers that have undergone therapeutic selection remains to be determined.

**Figure 2:**
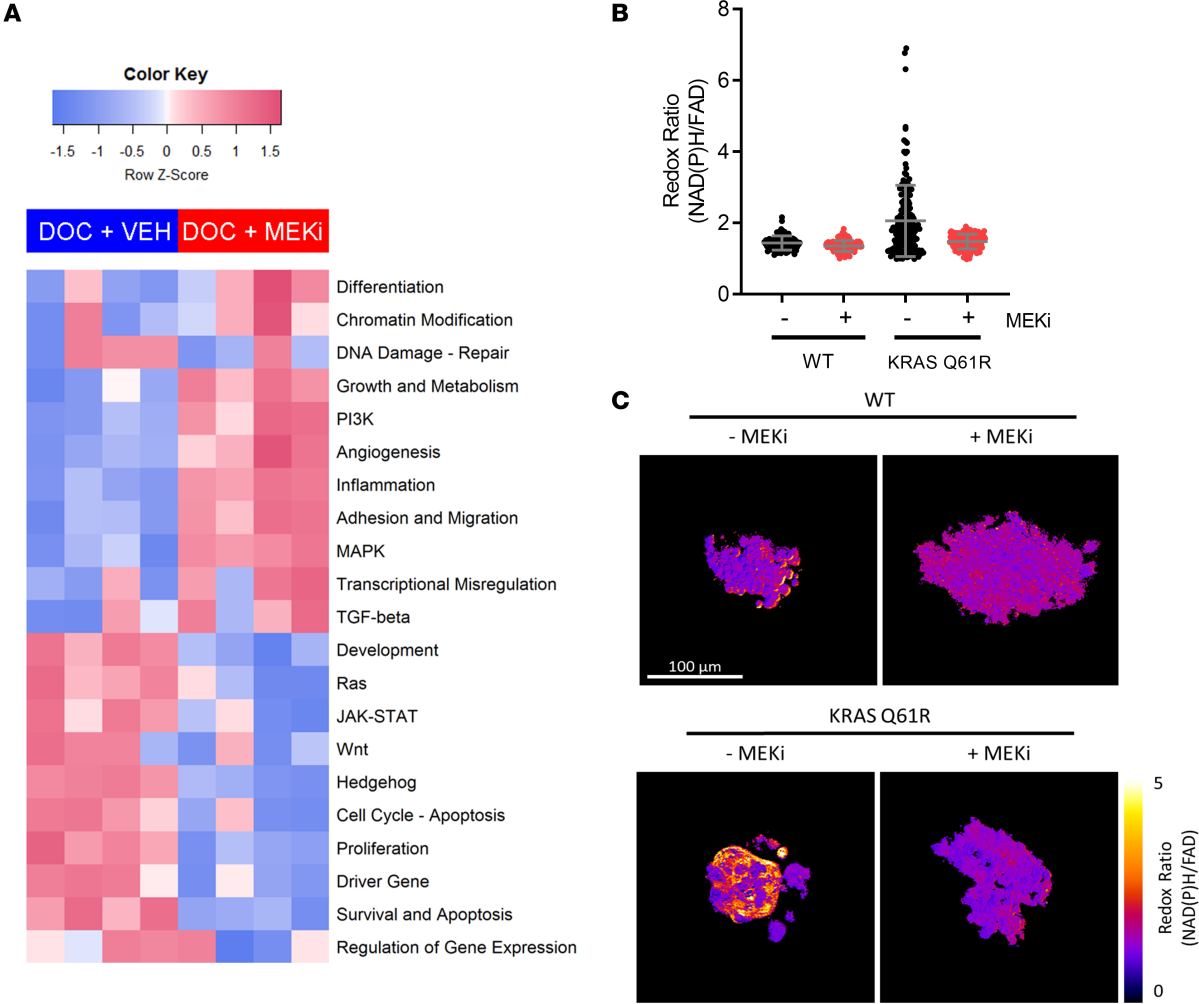
Metabolic and inflammatory phenotypes result from KRAS activation and are abrogated by MEK inhibition. (**A**) Gene set analysis of BCM-2277 tumors treated with AC**→**docetaxel + vehicle or AC**→**docetaxel + trametinib. Signature scores were calculated and visualized using the nSolver package (NanoString). (*n* = 4.) (**B**) Single-cell–level optical metabolic imaging of tumor organoids derived from BCM-2147 (*KRAS^WT^*) and BCM-2277 (*KRAS^Q61R^*) tumors, treated for 72 hours in the presence of 50 nM trametinib or DMSO control. (*n* > 75.) (**C**) Representative metabolic imaging of organoids from **B**.

To gain additional insights into the molecular phenotypes altered by MEK inhibition in the BCM-2277 *KRAS^Q61R^* model, we performed differential gene set analysis using the RNA data generated by 3D Biology analysis. Significantly altered gene sets in tumors treated with MEKi suggested suppression of proliferation and suppression of MEK and Ras/MAPK pathway activation, as expected based on our in vivo study (Figure 2A). Interestingly, MEKi treatment also reduced expression of gene sets representing inflammation and growth/metabolism in BCM-2277, suggesting Ras/MAPK signaling drives multiple oncogenic pathways in response to *KRAS* mutation (Figure 2A). Further analysis of (phospho)-proteins altered with MEKi also demonstrated suppression of p-AMPKα, a known regulator of metabolic homeostasis (Table 1).

To evaluate the possibility of a MEK-dependent metabolic phenotype, we performed optical metabolic imaging (OMI) (28, 29) of tumor organoids derived from BCM-2147 and BCM-2277 PDXs, grown in the presence or absence of MEKi for 72 hours. OMI is a multiphoton fluorescence microscopy technique that quantifies the endogenous fluorescence intensity of cellular NAD(P)H and flavin adenine dinucleotide (FAD) in single cells. The optical redox ratio, defined as the ratio of NAD(P)H fluorescence intensity to that of FAD, is sensitive to shifts in metabolic pathways that oxidize/reduce these coenzymes and reflects the overall redox state of the cell (28, 30, 31). Thus, inhibition of glycolysis by MEKi would be reflected in a decrease in the redox ratio. Single-cell analysis of tumor organoids across hundreds of cells in each organoid revealed a shift in the redox ratio (corresponding to enhancement of glycolysis versus oxidative phosphorylation) in the BCM-2277 *KRAS^Q61R^* model that was completely reversed by MEK inhibition (Figure 2, B and C). OMI studies in TNBC cell lines (MDA-231 [*KRAS^G13D^*], SUM159PT, and BT549) revealed that MEKi had little effect on glycolytic phenotypes in 2D culture (Supplemental Figure 5A); however, in 3D organoids of the same cell lines, MEKi resulted in a marked effect on the redox ratio, particularly in the *KRAS*-mutant MDA-231 cells (Supplemental Figure 5, B–D). Furthermore, a previously published signature of Ras/MAPK activation exhibited a positive correlation with the glucose transporter *SLC2A3* (GLUT3) and a nonsignificant trend toward positive correlation with the glucose transporter *SLC2A1* (GLUT1), both of which are necessary for glycolysis, in publicly available TNBC The Cancer Genome Atlas (TCGA) data (Supplemental Figure 6) (32). Thus, the metabolic phenotypes (i.e., Warburg effect) induced by mutant *KRAS* (12) appear to be primarily a result of MEK activation, and specific to 3D growth conditions, suggesting a role for hypoxia and/or matrix constituency. These glycolytic phenotypes have been reproducibly linked to chemoresistance (33–35), resistance to targeted inhibitors (36–38), and general immunosuppression via reduced T cell activation (33, 39). Furthermore, glycolytic function promotes tumor cell survival in hypoxic conditions, further contributing to an immunosuppressive tumor niche (40, 41).

### Ras/MAPK activation increases cytokine expression in TNBC PDXs and cell lines.

**Figure 3:**
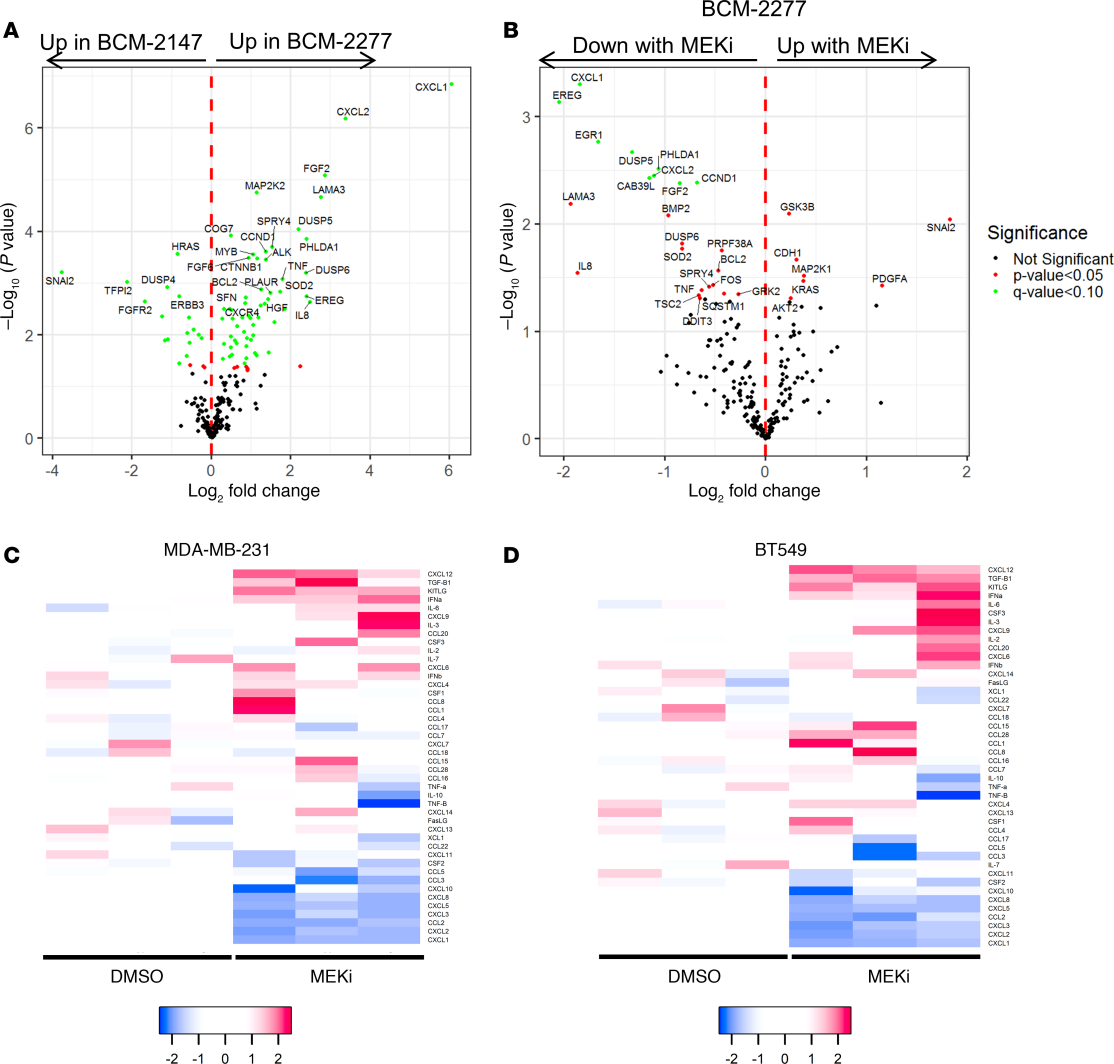
Unique transcriptional patterns associated with a rare KRAS mutation in TNBC PDXs. (**A**) Volcano plots of changes in gene expression between control group BCM-2277 and BCM-2147 PDX model samples. Genes are color-coded red if adjusted (FDR) *P* < 0.05, green if both FDR < 0.05 and log_2_ fold change > 1. (**B**) Volcano plots of changes in gene expression between control and trametinib-treated BCM-2277 samples. Genes are labeled as in **A**. (**C** and **D**) NanoString RNA analysis with a custom cytokine panel for relative gene expression (log_2_) in MDA-MB-231 (**C**) and BT549 (**D**) cells treated ± MEKi for 24 hours in vitro. (*n* = 3.)

Given the similarity in gene expression and mutational profile between the matched BCM-2147 and BCM-2277 PDXs, we asked what genes were differentially expressed between the 2 models in response to chemotherapy. We identified a distinct gene expression signature of primarily upregulated genes in the *KRAS^Q61R^* BCM-2277 model in response to chemotherapy treatment (Figure 3A). A substantial portion (9/32 altered at least 2-fold with *P* < 0.05) were also downregulated with the addition of MEKi to the combination chemotherapy regimen by comparing AC**→**T– and AC**→**TM-treated samples from the BCM-2277 model. These genes included myeloid-recruiting chemokines *CXCL1* and *CXCL2* (Figure 3B and Supplemental Figure 7A). A signature derived from these genes was also downregulated, albeit heterogeneously, with 4 or 24 hours of MEKi treatment (7) in TNBC cell lines (Supplemental Figure 7B). Interestingly, the signature was most affected in MDA-MB-231 cells (*KRAS^MUT^*) and SUM159PT cells (*HRAS^MUT^*). Thus, distinct gene expression patterns present in Ras/MAPK-activated TNBC can be reversed by MEK inhibition. Further evaluation of myeloid-associated chemokines and colony-stimulating factors (CSFs) using quantitative real-time polymerase chain reactions (qRTPCRs) demonstrated that *CXCL1*, *CXCL2*, and *CSF2* (another cytokine involved in myeloid trafficking/differentiation that was not included in the prior gene expression panel) are downregulated in response to MEKi treatment in a panel of TNBC cell lines, with *CSF2* expression being the most strongly and consistently downregulated (Supplemental Figure 7C). To more broadly ascertain the effects of MEKi on chemokines, MDA-MB-231 and BT549 cells were treated with or without MEKi and analyzed for expression of 44 cytokines and chemokines across 3 replicate treatment samples (Figure 3, C and D). Consistent with previous data, *CXCL1*, *CXCL2*, and *CSF2* were among the most strongly downregulated cytokines in response to MEKi treatment. Surprisingly, expression of the T lymphocyte–recruiting CXCL12 (42) was strongly increased in both cell lines tested, along with TGF-β1, KITLG, and IFN-α, suggesting additional immune-associated chemokine networks were altered by MEKi treatment (Figure 3, C and D).

### Myeloid recruitment to TNBC is mediated by Ras/MEK-dependent CXCL1/2 expression.

**Figure 4:**
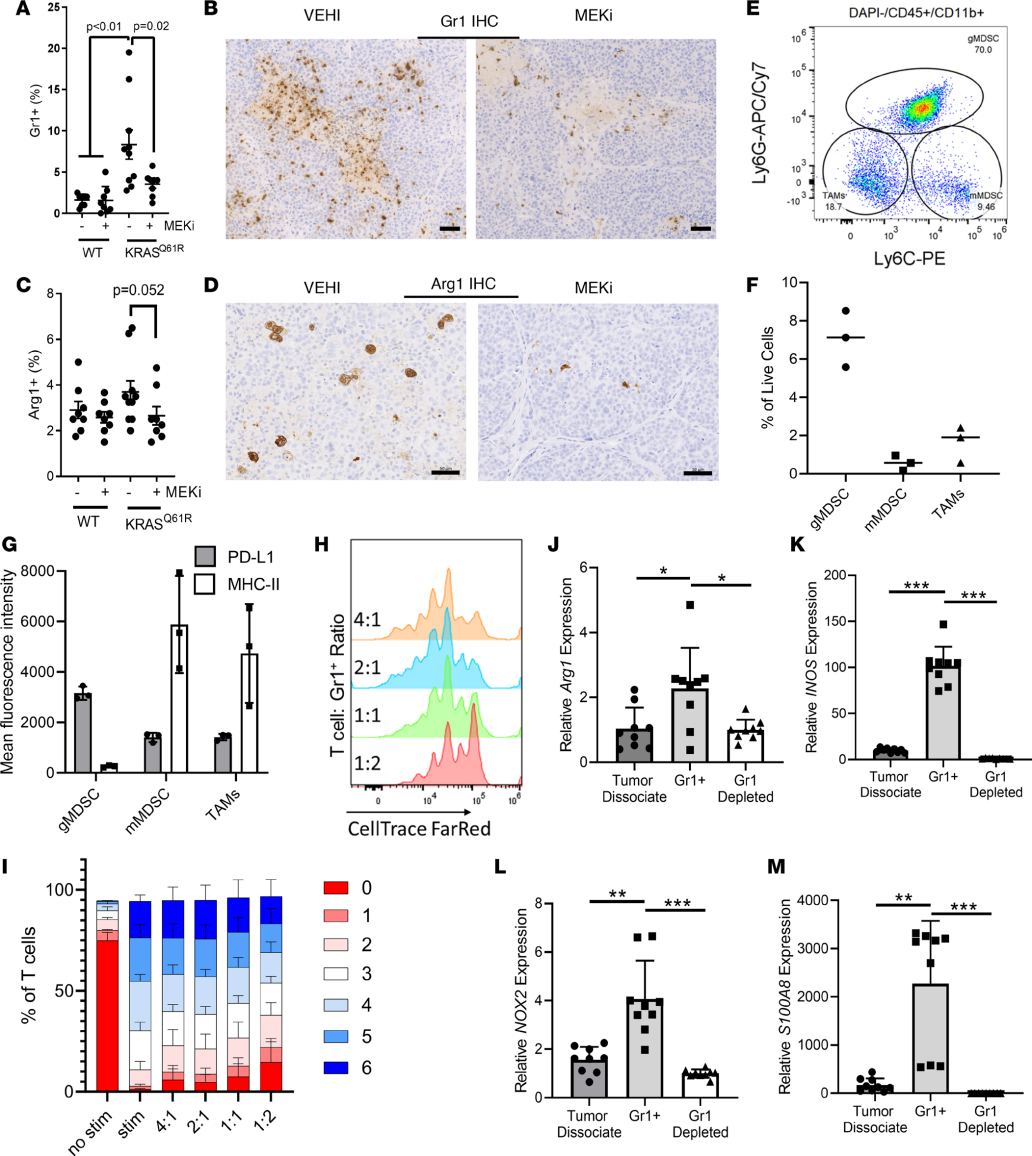
Myeloid recruitment to TNBC is mediated by Ras/MEK-dependent CXCL1/2 expression. (**A**) Quantification of Gr1^+^ myeloid cells in the tumor microenvironment in BCM-2147 (*KRAS^WT^*) and BCM-2277 (*KRAS^Q61R^*) tumors after treatment with AC/docetaxel + vehicle (VEHI) or AC/docetaxel + trametinib (MEKi). (*n* = 8–10.) Identified *P* values represent Tukey’s post hoc comparisons following 1-way ANOVA (*P* < 0.0001). (**B**) Representative images of Gr1^+^ cells from BCM-2277 VEHI- and MEKi-treated tumors (Scale bar: 50 μm). (**C**) Quantification of Arg1^+^ myeloid cells in the tumor microenvironment in BCM-2147 (*KRAS^WT^*) and BCM-2277 (*KRAS^Q61R^*) tumors after treatment with AC/docetaxel + vehicle (VEHI) or AC/docetaxel + trametinib (MEKi). (*n* = 8–10.) One-way ANOVA was nonsignificant. *P* value represents a 2-sample, 1-tailed *t* test between the MEKi and control arms of the *KRAS^Q61R^* model. (**D**) Representative images of Arg1^+^ cells from BCM-2277 VEHI- and MEKi-treated tumors. (Scale bar: 50 μm.) (**E**) Flow cytometry analysis of Ly6C/Ly6G expression in untreated BCM-2277 (*KRAS^Q61R^*) tumors, gated on DAPI^–^CD45^+^CD11b^+^. mMDSC, monocytic MDSC. (**F**) Relative percentages of 3 populations of myeloid cells as defined in **E** among 3 tumors. (**G**) Mean fluorescence intensity of PD-L1 and MHC-II (IA-IE) in the 3 myeloid populations in **E**. (**H**) T cell proliferation after 72 hours of coculture with Gr1^+^ cells and CD3/CD28 bead stimulation measured by CellTrace Far Red fluorescence. (**I**) Distribution of T cell proliferation in 72-hour cocultures with Gr1^+^ cells across 3 independent experiments. (**J**–**M**) RNA isolated from tumor dissociates, Gr1^+^ cells, and Gr1-depleted dissociates was probed for *Arg1*, *INOS*, *NOX2*, and *S100A8* by qRTPCR (*n* = 3).

CXCL1/2/8, all chemokines that were coordinately regulated by Ras/MAPK activity in PDX models and TNBC cell lines, bind CXCR2 to recruit myeloid cells, including neutrophils and MDSCs, to sites of inflammation. To determine the effects of MAPK signaling on the recruitment of immunosuppressive myeloid cells, we performed immunohistochemistry (IHC) for Gr1 (Ly6C/Ly6G) in tumors from mice treated according to the schema in Figure 1B. Given the marked differences between BCM-2147 and BCM-2277 tumors and the propensity for larger tumors to have increased levels of necrosis, necrotic regions were excluded from IHC scoring. We observed a striking enrichment of Gr1^+^ myeloid cells in BCM-2277 (*KRAS^Q61R^*), which was markedly reduced with MEKi (Figure 4, A and B). Analysis of arginase 1 (Arg1) expression by IHC in myeloid cells in situ demonstrated a similar but nonsignificant trend (Figure 4, C and D). Flow cytometry of myeloid populations in untreated BCM-2277 (*KRAS^Q61R^*) tumors demonstrated the majority of CD11b^+^GR1^+^ cells to be Ly6G^hi^Ly6C^lo^, likely representing a granulocytic MDSC (gMDSC) population, which was high in programed cell death ligand 1 (PD-L1) expression and low in MHC-II expression (Figure 4, E–G). To verify that these were suppressive myeloid cells, Gr1^+^ cells were isolated by affinity column before coculture with CD3/CD28-stimulated and fluorescently labeled T cells at various dilutions for 72 hours before flow cytometry analysis for cell proliferation (Supplemental Figure 8). An increased proportion of slowly dividing T cells were observed in the 1:1 and 1:2 (T cell/Gr1^+^) cocultures, demonstrating functional T cell suppression by Gr1^+^ tumor-derived MDSCs (Figure 4, H and I). Furthermore, these Gr1^+^ cells exhibited marked enrichment for suppressive myeloid genes, including *Arg1*, *INOS*, *NOX2*, and *S100A8*, compared with initial tumor dissociates or Gr1-depleted dissociates (Figure 4, J–M). Taken together, these findings suggest that MAPK activation in BCM-2277 correlates with increased MDSC tumor recruitment.

### Systemic MEKi or CXCR2i treatment reduces suppressive gMDSC accumulation within BCM-2277 tumors.

**Figure 5:**
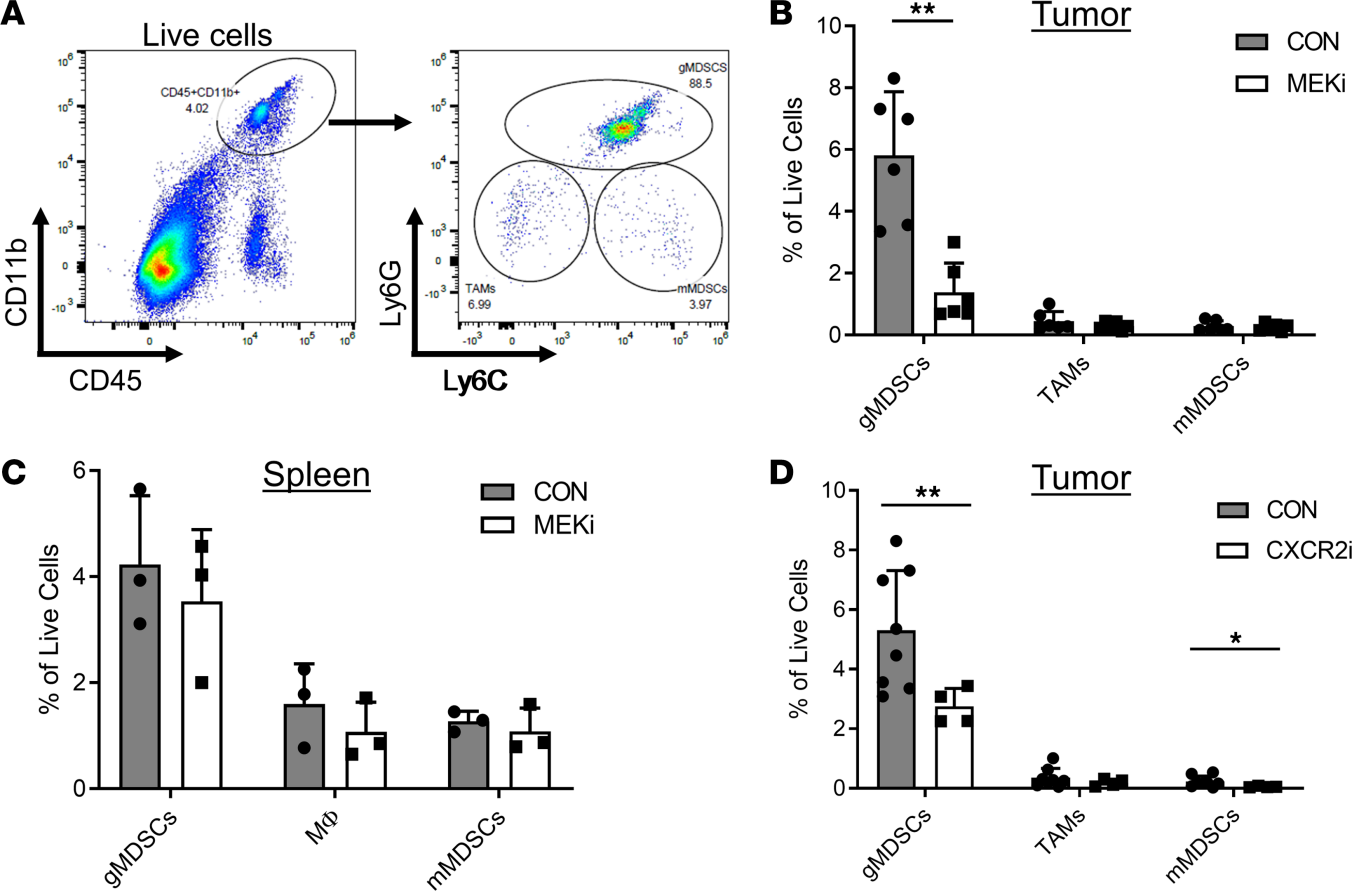
Inhibition of MEK or CXCR2 reduces gMDSC recruitment to tumors. (**A**) Flow cytometry gating strategy for MDSCs and macrophages. (**B**) BCM-2277 tumor–bearing mice were treated daily with MEKi before flow cytometry analysis of MDSCs and tumor-associated macrophages (TAMs) within tumor dissociates. (*n* = 6.) (**C**) Flow cytometry analysis for MDSCs and macrophages within the spleens of MEKi-treated mice. (**D**) BCM-2277 tumor–bearing mice were treated daily with CXCR2i (SB225002) before flow cytometry analysis of MDSCs and TAMs within tumor dissociates. (*n* = 8 CON, and *n* = 4 CXCR2i.) *P < 0.05; **P < 0.01.

To further explore whether the reduced Gr1^+^ and Arg1^+^ cells visualized by IHC were a result of MEKi treatment and not induced by synergistic or chemotherapy-dependent effects, we treated BCM-2277 tumor–bearing mice daily with or without MEKi for 7 days, without any chemotherapy, before flow cytometry analysis of CD45^+^CD11b^+^ cells for Ly6G or Ly6C expression. Importantly, CD45^+^CD11b^+^Ly6G^hi^Ly6C^lo^ cells, representing the gMDSC population, were decreased from approximately 6% to 1% of live cells within tumors treated with MEKi, but no changes were observed in CD45^+^CD11b^+^ populations within the spleen, suggesting that myeloid cell recruitment rather than differentiation was affected by MEK activity (Figure 5, A–C). Given this relatively high degree of myeloid cell infiltration, it is possible that a small portion of the previously described MAPK signaling and glycolytic activation within PDX tumors was due to myeloid cell population changes. To gain further insight on the molecular mechanism mediating gMDSC recruitment to BCM-2277 tumors, we then treated a cohort of tumor-bearing mice with an inhibitor of CXCR2, the receptor through which CXCL1 and CXCL2 signal. Systemic CXCR2i treatment for 7 days decreased both gMDSC and monocytic MDSC accumulation within tumors (Figure 5D). Importantly, in the absence of an adaptive immune response, the inhibition of CXCR2^+^ cell recruitment to the tumor exhibited a nonsignificant trend toward reduced tumor growth, suggesting that the loss of CXCR2^+^ cell recruitment was not sufficient to rescue MEKi-induced tumor suppression (data not shown). Moreover, these data suggest that MEKi and CXCR2i both affect myeloid cell recruitment to tumors and that CXCL1/CXCL2 mediate MEKi-induced effects on myeloid cell populations within BCM-2277 tumors.

### CXCR1/2 ligands are associated with Ras/MAPK transcriptional activity in breast cancer cell lines and tumors.

**Figure 6:**
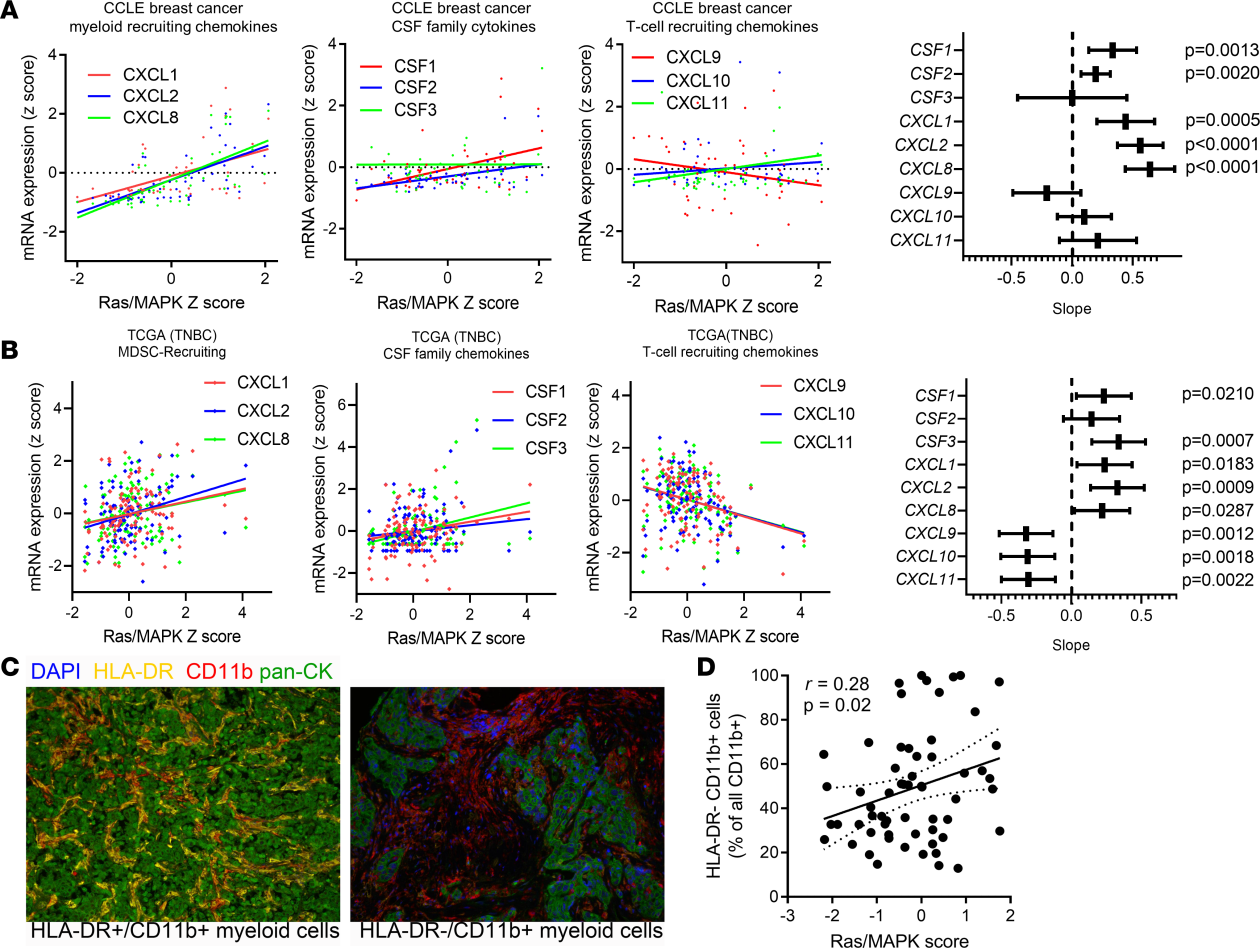
Association of MEK activation with CXCR1/CXCR2 ligands in cancer cell lines and human breast tumors. (**A**) Correlation of select cytokine mRNAs with the Ras/MAPK pathway activation gene signature score across 50 breast cancer cell lines in the CCLE. *CXCL1*, *CXCL2*, *CSF1*, *CSF2*, and *CXCL8* gene expression were all significantly associated with Ras/MAPK activity (*P* < 0.0001 for all), while CXCR3 (T cell–recruiting) chemokines were not associated with Ras/MAPK activity. (**B**) CXCR1/2 (MDSC-recruiting) chemokines were positively associated with Ras/MAPK activation in human TNBC (TCGA). CSF family members 1 and 3 were positively associated with Ras/MAPK activation in human TNBC (TCGA). *CXCL9*, *CXCL10*, and *CXCL11* (CXCR3 ligands/T cell–recruiting chemokines) were negatively associated with Ras/MAPK activation in human TNBC (TCGA). (**C**) Representative quantitative immunofluorescence analysis for HLA-DR (shown in yellow), CD11b (red), and pan-cytokeratin (green) with DAPI as nuclear counterstain. Original magnification, ×200. (**D**) Correlation of Ras/MAPK transcriptional score versus CD11b^+^HLA-DR^–^ (immunosuppressive myeloid cells) expressed as a percentage of all CD11b^+^ cells across 61 TNBCs after neoadjuvant chemotherapy (6, 21). Data were assessed in tissue microarray format, using the average cell number across 3 independent cores per patient sample.

To evaluate the relevance of the association between Ras/MAPK activation and MDSC-recruiting chemokines to human disease, we first explored the Cancer Cell Line Encyclopedia (CCLE database). Across cell lines from all tumor types, we observed enrichment of *CXCL1* expression in KRAS^MUT^ cell lines versus KRAS^WT^ cell lines (Supplemental Figure 9). Interestingly, the same effect was not observed in NRAS^MUT^ cell lines, reflecting possible differences in *RAS* isoform biology. Refining the data to all breast cancer cell lines and using a previously published signature of Ras/MAPK activation (43) because of the low preponderance of activating *KRAS* mutations in breast cancer, we found strong positive associations of transcriptional activation of the Ras/MAPK pathway with MDSC-recruiting CXCR2 ligands (*CXCL1/2/8*) but not T cell–recruiting CXCR3 ligands (*CXCL9/10/11*) (Figure 6A).

To examine the relevance of these findings to human breast cancer, we analyzed breast cancers in TCGA data set (44) using cBioPortal (45). The Ras/MAPK transcriptional signature was highly associated with expression of *CXCL1/2/8* and *CSF1/2/3* across TNBC/basal-like breast tumors, while T cell–recruiting CXCR3 chemokines were negatively associated with Ras/MAPK activity (Figure 6B). Moreover, these correlations between myeloid-associated or T cell–recruiting chemokines were also statistically significant when measured across more than 1000 breast cancer samples from TCGA independent of clinical subtypes, suggesting that Ras/MAPK activity may also regulate chemokine production in other forms of breast cancer (Supplemental Figure 10). In a microarray data series of 201 genetically engineered mouse models of breast cancer, similar associations for *Cxcl1* were also identified (Supplemental Figure 11). Thus, activation of the Ras/MAPK pathway, either through oncogenic KRAS activation, or other mechanisms, may drive MDSC recruitment in breast cancers in both mice and humans. While Ras/MAPK score was not associated with poor survival in TCGA TNBC/basal-like breast cancer subset, previous work by our group demonstrated that increased Ras/MAPK score correlated with poor prognosis in residual disease after neoadjuvant chemotherapy in a TNBC cohort (21). To confirm the relationship between MDSC recruitment and Ras/MAPK activity at the protein level in human TNBC, we performed multiplexed immunofluorescence for the MHC-II protein human leukocyte antigen DR isotype (HLA-DR), pan-cytokeratin, and CD11b in a tissue microarray comprising 61 cases of TNBC after neoadjuvant chemotherapy (all residual disease) (6, 21) where mRNA expression data for the Ras/MAPK transcriptional signature (10, 43) were available (Figure 6C). Using HLA-DR^–^ (MHC-II^–^) CD11b^+^ cells as a marker of immunosuppressive myeloid cells, we confirmed a positive correlation (*P* = 0.02; *r* = 0.28) between Ras/MAPK signaling and suppressive myeloid cell presence in TNBC (Figure 6D). Given that MEKi elicit immunologic effects as well as direct antitumor effects (10, 46) and are actively being combined with immune checkpoint inhibitors in breast cancer, suppression of MDSC recruitment via transcriptional inhibition of *CXCL1/2/8* may be a novel mechanism of combinatorial activity.

## Discussion

Our work has focused on the study of paired breast cancer PDXs before and after the development of chemotherapy resistance to identify targetable, *KRAS* mutant–associated signaling mechanisms that promote tumor progression and alter the tumor immune microenvironment. Specifically, 3D Biology analysis of PDX tumors treated with a combination of chemotherapy and MEKi identified immune and metabolic pathways that were inhibited by MEKi treatment. Using OMI (28, 29), we observed glycolytic reduction/oxidation changes in organoids derived from PDXs that corresponded to the acquisition of *KRAS^Q61R^* and that were reversed completely by MEKi. Furthermore, we identify recruitment of immunosuppressive Gr1^+^ myeloid cells via tumor cell expression of *CXCL1*, *CXCL2*, and *CXCL8* (CXCR2 ligands) expression, which were substantially reduced with MEKi or CXCR2 blockade. These findings were validated across 115 cases of human TNBC, where we observed that Ras/MAPK transcriptional patterns demonstrate correlations with expression of myeloid-recruiting chemokines and inverse correlations with T cell–recruiting chemokines.

The activation of the Ras/MAPK pathway has previously been implicated in breast cancer and other tumor types as a source of immunosuppressive signals (11). Our recent work has identified suppression of antigen presentation as an additional mechanism whereby Ras/MAPK suppresses antitumor immunity, despite its proliferative effects on naive T cells (10, 47). Recent studies of *Kras*-driven murine models have found associations between tumors induced by activated Kras and secretion of CXCR2-binding cytokines (13, 48, 49), although these studies have focused on the role of Kras-induced NF-κB pathway activity, rather than MEK. Allegrezza et al. demonstrated that MEKi treatment could reduce tumor-infiltrating MDSCs in Kras-mutant models; however, the mechanism was not defined within that study (46). Recent work in Kras-mutant colorectal cancer models suggests that Kras regulates MDSC recruitment via IRF2 and tumor cell secretion of CXCL3 (50). While the mechanism seems to vary between tissues, the connection between increased Ras/MAPK activity and MDSC recruitment appears to be conserved, because our data suggest that MEK activation downstream of oncogenic KRAS drives MDSC recruitment to breast tumors.

MEK activation and MDSC recruitment may be targetable for some breast cancer patients; however, MEK inhibition did not completely reverse suppressive myeloid cell recruitment in vivo, suggesting additional pathways may contribute to this phenotype. Furthermore, a high degree of Gr1^+^ cells was not observed in the other 3 models tested, despite HBCx1 demonstrating moderate ERK activation and marginal sensitivity to MEKi. This finding could be explained by the need for additional activation of NF-κB resulting from the consistent signaling induced by activated KRAS (46, 48, 49) in the BCM-2277 model. Several studies have attributed effects of oncogenic KRAS to this pathway, and chemokine/secretory phenotypes such as expression of CXCL1/2/8 have been shown to rely on NF-κB pathway activation.

Nonetheless, our findings directly implicate activation of MEK in the recruitment of suppressive myeloid cells and in the generation of glycolytic phenotypes that may further feed into immunosuppressive tumor microenvironments. Because early-phase trials show promising activity in TNBC with combinations of MEKi and taxanes (18), the effects on MDSC recruitment and metabolic phenotypes should be explored as potential biomarkers associated with outcome. Furthermore, new clinical trials are now being initiated using MEKi, taxanes, and anti–PD-L1 antibodies, which unleash suppressed T cells in the microenvironment (51). Given the role of MDSCs in T cell suppression (52), the blockade of MDSC recruitment coupled with therapies that reinvigorate T cell responses may be more successful therapeutically in these trials.

## Methods

### PDXs.

PDX models were established and characterized as previously reported (BCM-4013, BCM-2147, BCM-2277 [ref. 22], and HBCx1 [ref. 53]). For propagation, 2 × 2 mm tumor samples were serially passaged by number 4 mammary fat pad transplantation under general anesthesia in 6-week-old female athymic NCr nude/nude mice (Envigo, formerly Harlan). To reduce the likelihood of genetic drift, only PDXs in the first 5 passages were used for study. Mice bearing tumor sizes at least 150 mm^3^ were randomized to treatment with doxorubicin (2 mg/kg/wk i.p.) and cyclophosphamide (100 mg/kg/wk i.p.) for 2 weeks, followed by docetaxel (20 mg/kg/wk i.p.) and oral gavage vehicle or docetaxel + trametinib (1 mg/kg/d by mouth). Trametinib dosing was completed on a per weight basis, building on a previous pharmacokinetic study in rodents (54). At the end of 4 total weeks of treatment (2 weeks of doxorubicin + cyclophosphamide and 2 weeks of docetaxel ± trametinib), mice were euthanized, and residual tumors were resected for analysis. During the study, tumor diameters were measured using calipers 3 times per week, and volume in mm^3^ was calculated with the following formula: volume = width^2^ × length/2.

### Immunoblotting.

Immunoblotting was performed as previously described (55). Briefly, tumor fragments were homogenized in 1× RIPA buffer lacking SDS detergent (50 mM Tris pH 7.4, 150 mM NaCl, 1.0% NP-40, 0.5% deoxycholic acid, 1 mM EDTA, 1 mM EGTA, 5 mM sodium pyrophosphate, 50 mM NaF, 10 mM β-glycerophosphate) with added phosphatase inhibitors (PhosSTOP, Roche) and protease inhibitors (cOmplete, Roche) using a QIAGEN Tissue Lyser. Lysates were adjusted to 0.1% SDS, followed by 30 minutes’ incubation on ice. Lysates were centrifuged at 13,000 *g* for 15 minutes at 4°C. Protein concentrations of the lysates were determined by BCA Protein Assay (Thermo Fisher Scientific). Samples were separated by 10% SDS-PAGE and transferred to nitrocellulose membranes. Membranes were blocked with 5% nonfat dry milk or 5% BSA in tris-buffered saline with 0.1% Tween-20 for 1 hour at room temperature and then incubated overnight at 4°C with the appropriate antibody in blocking buffer as indicated. Following incubation with appropriate HRP-conjugated secondary antibodies, proteins were visualized using an enhanced chemiluminescence detection system (Thermo Fisher Scientific). This study was performed using the following antibodies: calnexin (SC11397; Santa Cruz Biotechnology), ERK1/2 (#9102), p-ERK1/2 (#4370), and p-S6 (#5301), all of which were purchased from Cell Signaling Technology along with the PathScan Multiplex Western Cocktail I: p-p90RSK, p-AKT, p-ERK1/2, p-S6, and Rab11 were also from Cell Signaling Technology.

### NanoString 3D analysis.

Two 5 μm sections from FFPE blocks were cut onto slides for simultaneous DNA, RNA, and protein analysis. The expression of 26 proteins and 192 RNAs as well as the analysis of 104 clinically actionable single nucleotide variants (SNVs) were simultaneously measured using the nCounter Vantage 3D Solid Tumor Assay for FFPE (NanoString Technologies) reagent using NanoString protocols. Briefly, for protein analysis, sections were subjected to deparaffinization and rehydration followed by epitope retrieval with pH 6.0 citrate buffer and overnight incubation with the DNA-labeled antibody mix at 4°C. Following washes to remove nonspecific antibodies, the slides were placed on an ultraviolet transilluminator for 3 minutes to release the photo-cleavable DNA tags. The DNA tags were denatured and then hybridized to target specific fluorescent barcodes. For RNA expression and SNV analysis, RNA and DNA were purified from a single 5 μm section using the QIAGEN AllPrep kit. DNA mutational analysis was conducted using a commercial multilocus targeting assay kit, the nCounter Vantage 3D DNA SNV Solid Tumor Panel, on an nCounter MAX Analysis System. The assay uses multiplex PCR to amplify 40 human genomic loci from 25 genes that are frequently mutated in solid tumors. Following the PCR step, the DNA amplicons are interrogated by specialized DNA probes designed to specifically hybridize to short nucleotide variants (single- and dinucleotide substitutions and insertions or deletions of up to 18 nucleotides) and additional probes that specifically hybridize to the GRCh37/hg19 reference sequence that corresponds to the position of each assayed variant. After hybridization, stable complexes are immobilized and counted on the nCounter system. For RNA expression analysis, 100 ng of RNA was hybridized to target specific fluorescent barcodes. The hybridized samples for DNA, RNA, and protein analysis were simultaneously analyzed on the NanoString nCounter MAX Analysis system followed by data processing and primary analysis with the NanoString nSolver data analysis software.

### Cell lines and treatment.

Human breast cancer cell lines MDA-MB-231 (DMEM + 10% fetal bovine serum; FBS), BT549 (RPMI + 10% FBS), HCC38 (RPMI + 10% FBS), MD-MB-436 (DMEM + 10% FBS), HCC70 (RPMI + 10% FBS), HCC1143 (RPMI + 10% FBS), HCC1954 (RPMI + 10% FBS), and SUM159PT (DMEM/F-12 + 5% FBS + 0.05 mg/mL hydrocortisone) were obtained from ATCC and routinely tested for mycoplasma contamination. In vitro trametinib treatments were completed at 50 nM, which is in range with C_max_ concentrations detected in preclinical and clinical pharmacokinetic studies (54, 56).

### Flow cytometry.

Cells were washed in phosphate-buffered saline (PBS) and harvested with Accutase (MilliporeSigma, SCR005) for 10 minutes at room temperature. Dissociated cells were washed once in flow staining buffer (PBS + 1% FBS) and incubated with respective flow antibodies at 4°C for 20 minutes in the dark. Flow cytometry was performed using the following antibodies: CD45/AF488 (BioLegend clone 30-F11, 1:500), Ly6C/PE (BioLegend clone HK1.4, 1:250), Ly6G/APC-Cy7 (BioLegend clone 1A8,1:250), CD11b/PE-Cy7 (BioLegend clone M1/70,1:250), CD274 (PD-L1)/APC (BioLegend clone 10F.9G2, 1:250), and mouse MHC-II (I-A/I-E)/BV711 (BioLegend clone M5/114.15.2, 1:500). DAPI was used as a viability dye for dead cell exclusion. Samples were analyzed on an Attune NxT flow cytometer (Life Technologies, Thermo Fisher Scientific).

### IHC.

FFPE sections from human PDX tumors grown in athymic *nu/nu* mice were sectioned for IHC analysis. Slides were placed on the Leica Bond Max IHC stainer. All steps besides dehydration, clearing, and coverslipping were performed on the Bond Max. Slides were deparaffinized. Heat-induced antigen retrieval was performed on the Bond Max using the manufacturer’s Epitope Retrieval 2 solution for 20 minutes. Slides were incubated with anti–neutrophil marker (Ly6G/C [Gr1], ab2557, Abcam) for 1 hour at a 1:2000 dilution and then incubated in a rabbit anti-rat secondary (BA-4001, Vector Laboratories, Inc.) for 15 minutes at a 1:200 dilution; the Bond Polymer Refine Detection system was used for visualization. For Arg1 (Santa Cruz Biotechnology, sc-18351; 1:400), after incubation overnight at 4°C, citrate buffer pH 6 was used for antigen retrieval using a Biocare Medical Decloaking Chamber, secondary antibody goat/HRP (Dako, P0449) for 30 minutes at a 1:100 dilution, and DAB as a chromogen, counterstained with hematoxylin. Slides were then dehydrated, cleared, and coverslipped. Gr1^+^ and Arg1^+^ cells were scored by a research pathologist as average positive cells per high-power field across at least 10 fields.

### Tumor cell dissociation and in vitro T cell assay.

Splenocytes were isolated from 8-week-old BALB/cAnNCr mice (Envigo, formerly Harlan) and labeled with CellTrace Far Red dye for 30 minutes in serum-free PBS (Thermo Fisher Scientific, 1:1000). BCM-2277 tumors were harvested from mice after reaching more than 500 mm^3^ for tumor cell dissociation (in Serum-Free RPMI from Gibco, Thermo Fisher Scientific, 2.5 mg/mL, and Collagenase 3, 62.5 μg/mL, from Worthington) using the gentleMACs Octo dissociator (Miltenyi Biotec) default tumor protocol for 30 minutes at 37°C under constant agitation. The tumor dissociate was then passed through a 40 μm filter and washed with 20–30 mL of PBS. MDSCs were isolated using the Myeloid-Derived Suppressor Cell Isolation Kit (Miltenyi Biotec, mouse) according to the manufacturer’s protocol. The Gr1^+^ enriched cells from this isolation were then counted using trypan blue and a LUNA-II automated cell counter (Logos Biosystems) and plated with negatively selected labeled T cells (Pan T Cell Isolation Kit, Miltenyi Biotec) isolated from WT BALB/c mice (Envigo, formerly Harlan), for a total of 30,000 cells per well in RPMI medium (1% HEPES, 50 μM BME, 10 ng/μL mouse IL-2, BD Biosciences), in a round-bottom 96-well cell culture plate (Corning). After 72 hours of CD3/CD28 Dynabead (Thermo Fisher Scientific) stimulation, the cells were processed for flow cytometry analysis using an Attune NxT flow cytometer (Life Technologies, Thermo Fisher Scientific).

### Multiplexed immunofluorescence.

Fluorescence IHC staining, imaging, and analysis were performed as described previously (57), where rabbit anti-IDO1 (SP260, 1:500 dilution, Abcam) was detected with Opal 620 (1:200 dilution, Akoya Biosciences) followed by chemical quenching of residual HRP using 100 mM benzhydrazide with 50 mM hydrogen peroxide. Mouse anti–HLA-DR (TAL.1B5, 1:500 dilution, Agilent) was detected with Opal 650 (1:200 dilution, Akoya Biosciences), and then all primary and secondary antibodies were removed via microwave to allow staining with rabbit anti-CD11b (EY1345Y, 1:500 dilution, Abcam) detected with Opal 520 (1:200 dilution, Akoya Biosciences) followed by chemical quenching of residual HRP using 100 mM benzhydrazide with 50 mM hydrogen peroxide. Finally, mouse anti–pan-cytokeratin (AE1/AE3, 1:100, Agilent) was detected with Opal 570 (1:200 dilution, Akoya Biosciences), and DAPI was used to identify cell nuclei. Image analysis was performed using AQUA (Navigate BioPharma Services, Inc.) to determine the percentage of all DAPI^+^ cells that are CD11b^+^, HLA-DR^+^, IDO1^+^, CD11b^+^HLA-DR^+^, or CD11b^+^HLA-DR^–^ cells. The IDO1 channel was not used for analysis in the present study.

### qRTPCR.

RNA was harvested from cells using the Maxwell 16 automated workstation (Promega) and LEV simplyRNA Tissue Kit (Promega). RNA was then analyzed for concentration by a NanoDrop 2000 (Thermo Fisher Scientific) before cDNA synthesis using SensiFAST cDNA Synthesis Kit (Bioline, Meridian Bioscience, Inc.) with 1 μg of RNA per sample. cDNA and SsoAdvanced Universal SYBR Green Supermix (Bio-Rad) were then combined with target-specific primers on a CFX96 Touch Real-Time PCR Detection System (Bio-Rad). A heatmap was generated using GraphPad Prism 8. Primers included *CXCL1* Forward 5′-AGTCATAGCCACACTCAAGAATGG-3′ reverse 5′-GATGCAGGATTGAGGCAAGC-3′; *CXCL2* forward 5′-CTCAAGAATGGGCAGAAAGC-3′ reverse 5′-AAACACATTAGGCGCAATCC-3′; *CSF1* forward 5′-CACATGATTGGGAGTGGACA-3′ reverse 5′-TAATTTGGCACGAGGTCTCC-3′; *CSF2* forward 5′-CAGCCTCACCAAGCTCAAG-3′ reverse 5′-AATCTGGGTTGCACAGGAAG-3′; mARG1 forward 5′-GTGAAGAACCCACGGTCTGTG-3′ reverse 5′-CTGGTTGTCAGGGGAGTGTTG-3′; mINOS forward 5′-CACCTTGGAGTTCACCCAG-3′ reverse 5′-ACCACTCGTACTTGGGATGC-3′; mS100A8 forward 5′-GGAAATCACCATGCCCTCTA-3′ reverse 5′ -TCCTTGTGGCTGTCTTTGTG-3′; and mNOX2 forward 5′-ACTGCGGAGAGTTTGGAAGAG-3′ reverse 5′-GGTGATGACCACCTTTTGCTG-3′.

### Organoid generation and treatment.

Tumors were excised and immediately placed in chilled medium consisting of DMEM/F-12 (11330, Gibco, Thermo Fisher Scientific), 10 ng/mL EGF (AF-100-15, PeproTech), 5 μg/mL hydrocortisone (H0888, MilliporeSigma), 5 μg/mL insulin (I1882, MilliporeSigma), and 1% penicillin/streptomycin (15070, Gibco, Thermo Fisher Scientific). To generate organoids, tumors were rinsed with PBS and mechanically digested in media using scissors. The cell macrosuspension was mixed with Matrigel (356234, Corning) at a 1:2 ratio, and 100 μL of the resulting mixture was placed in 35 mm glass-bottom dishes (P35G-1.5-14-C, MatTek Corporation). Gels solidified at room temperature for 30 minutes, then at 37°C in an incubator for 1 hour before being overlaid with media. Organoids were treated for 72 hours with either 0.1% DMSO vehicle (D8418, MilliporeSigma) or 50 nM trametinib (Selleckchem). For breast cancer cell line imaging, 1 × 10^5^ cells were seeded 24 hours before drug treatment in 35 mm glass-bottom dishes. Cells were treated with 0.1% DMSO vehicle or 50 nM trametinib.

### Organoid OMI.

Autofluorescence images were acquired using an inverted custom-built multiphoton system (Bruker Fluorescence Microscopy), using either a ×40 oil immersion objective (1.3 NA, Nikon) or a ×40 water immersion objective (1.15 NA, Nikon). A titanium/sapphire laser (Chameleon Ultra II, Coherent or InSight DS+, Spectra-Physics) was used for 2-photon fluorescence excitation. NAD(P)H was excited using 750 nm light, and a 440/80 nm bandpass filter was used to collect its emission. FAD was excited at 890 nm and a 550/100 nm filter was used to collect its emission. A pixel dwell time of 4.8 μs and a total integration time of 60 seconds was used to collect 256 × 256. A GaAsP photomultiplier tube (H7422P-40, Hamamatsu Photonics) detected emitted photons.

After 72 hours of treatment, cell lines and organoids were imaged at 3–4 locations per dish for a total of 100–1000 cells imaged per treatment group for cell lines and 60–280 cells per group for organoids. NAD(P)H images were first acquired, followed immediately by an FAD image of the same field of view. All OMI experiments were repeated in triplicate.

CellProfiler was used to automatically identify individual cells and isolate average fluorescence intensity values for each (minus background and nuclear signals) (58). Optical redox ratio values were calculated for each cell by dividing the average intensity of NAD(P)H by the average intensity of FAD. Redox ratios for all cells in a treatment group were averaged together and normalized to control values within an experiment.

### Genomic databases.

Genomic and gene expression data from AACR GENIE, METABRIC (59), TCGA breast cancers (44), and CCLE (60) were accessed via the cBioPortal (45). Microarray data for MDA-MB-231, SUM159PT, and BT549 TNBC cell lines treated with MEKi (selumetinib) were accessed from GEO GSE41816, and generation of these data was previously described (7). The Ras/MAPK signature score was calculated from gene expression data as previously described (7, 21, 43). Genetically engineered mouse model microarray data were accessed and described as previously published (10, 61).

### Statistics.

Statistics were performed in GraphPad Prism or R (www.r-project.org). In data with 2 groups, 2-sample 1- or 2-tailed *t* tests were used. For analyses with more than 2 groups, significant differences were determined by 1-way ANOVA with a Tukey’s post hoc adjustment for multiple comparisons. For all multiple comparisons, statistical significance is noted by *P < 0.05, **P < 0.01, ***P < 0.001, and ***P < 0.0001. A *P* value of less than 0.05 was considered statistically significant. Bar graphs show mean ± SEM, unless otherwise stated in the figure legend. For correlations, the Pearson correlation coefficient was used to test significance of association.

### Study approval.

Athymic mouse experiments were approved by Vanderbilt University’s (VUMC) comprehensive Animal Care and Use Program (ACUP). The VUMC ACUP is registered with the United States Department of Agriculture (USDA registration 63-R-0129) and operates under a Public Health Service Animal Welfare Assurance Statement (PHS Assurance A3227-01). The VUMC ACUP has been accredited by the Association for the Assessment and Accreditation of Laboratory Animal Care, International, since 1967 (AAALAC file 000020) and most recently received continued full accreditation on June 21, 2017.

## Author contributions

DAF and JMB wrote the manuscript. DAF, RSC, and JMB edited the manuscript. DAF, JTS, PIEG, VS, PTD, SRO, SC, JGJ, MTL, JCC, MES, MCS, JB, JOB, and CV generated experimental materials and data. DAF, JTS, PIEG, MCS, GG, DH, and JMB analyzed the data.

## Supplementary Material

Supplemental data

## Figures and Tables

**Table 1:**
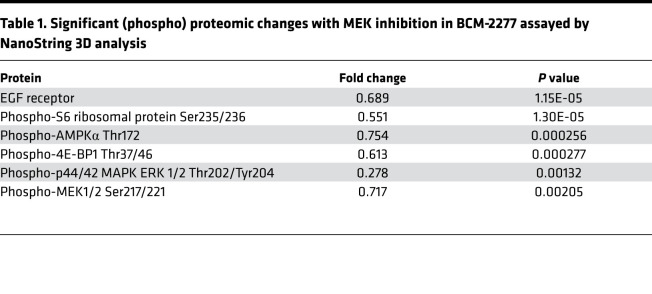
Significant (phospho) proteomic changes with MEK inhibition in BCM-2277 assayed by NanoString 3D analysis

## Version 1 07/07/2020

In-Press Preview

## Version 2 08/06/2020

Electronic publication
