# Supplementary material for: MEK activation modulates glycolysis and supports suppressive myeloid cells in TNBC
Source: JCI Insight. 2020 Aug 6;5(15):e134290. doi: 10.1172/jci.insight.134290 (PMC7455066; doi:10.1172/jci.insight.134290)
Supplement: Supplemental data [file jciinsight-5-134290-s028.pdf]

Supplementary Figure 1

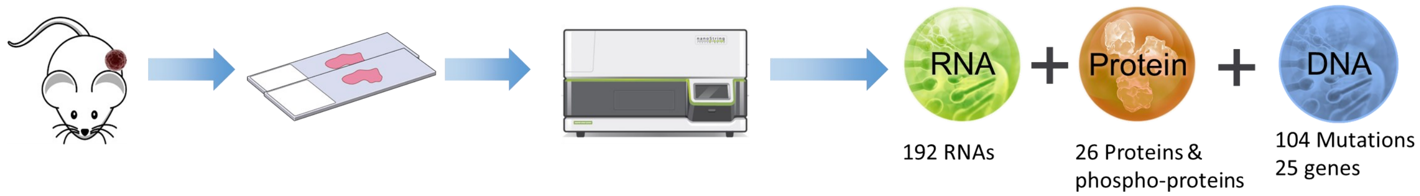

Supplementary Figure 1: NanoString simultaneous detection of RNA/DNA/(phospho)protein. Schematic for NanoString protein/RNA/DNA analysis from FFPE tissue sections

Supplementary Figure 2

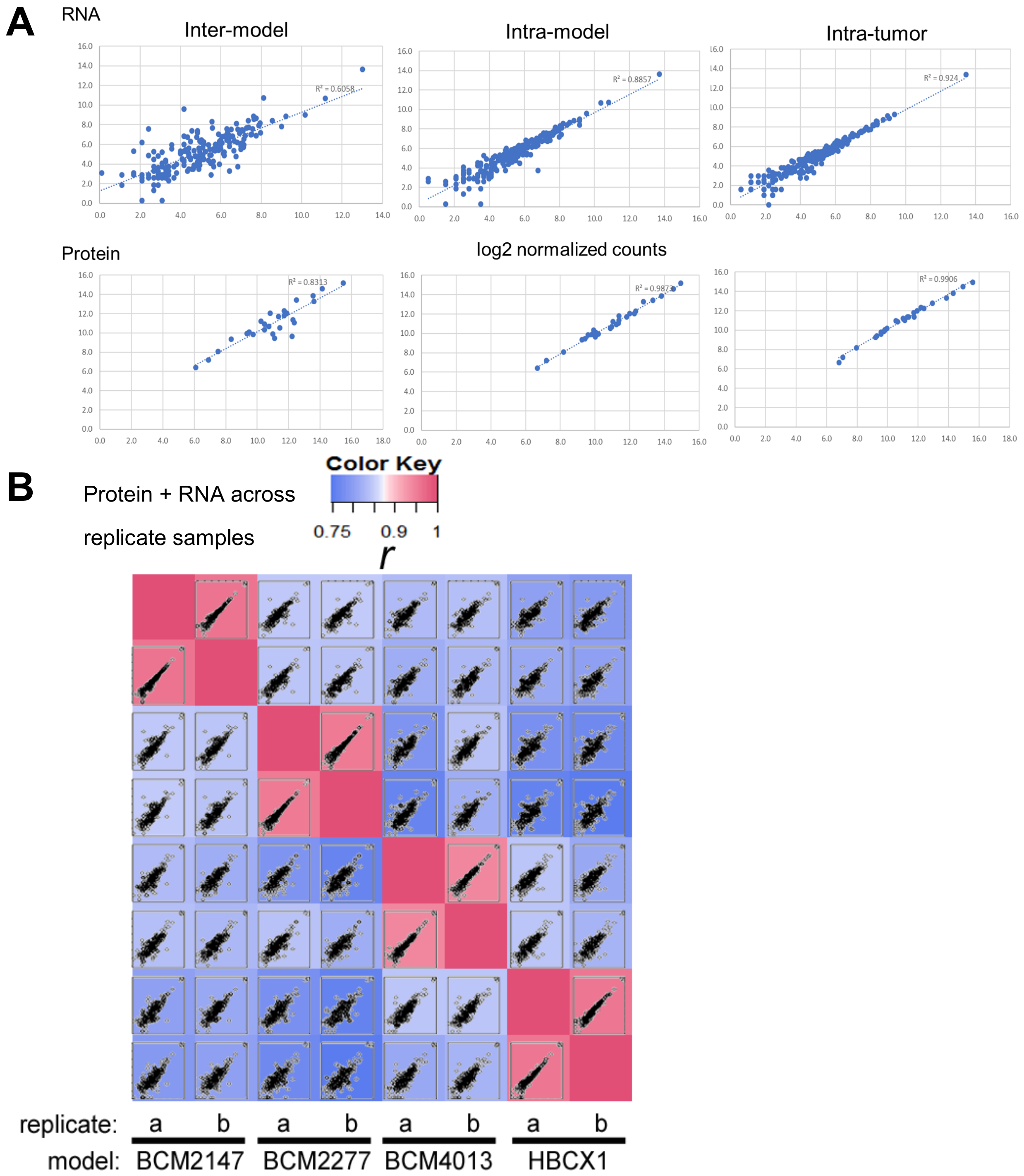

<u>Supplementary Figure 2: Cross model consistency and correlation of NanoString 3D data.</u> A) Upper: sample correlations of normalized RNA signal across models (Inter-model), across tumors in the same model (Intra-model) and in replicates from the same tumor (intra-tumor). Lower: sample correlations of normalized protein signal across models (Inter-model), across tumors in the same model (Intra-model) and in replicates from the same tumor (intra-tumor). B) Correlation matrix of protein and RNA data across replicate samples, tumors, and models. Only samples from each model that were replicated (i.e. the same tumor sample was analyzed twice for validation) are shown.

Supplementary Figure 3

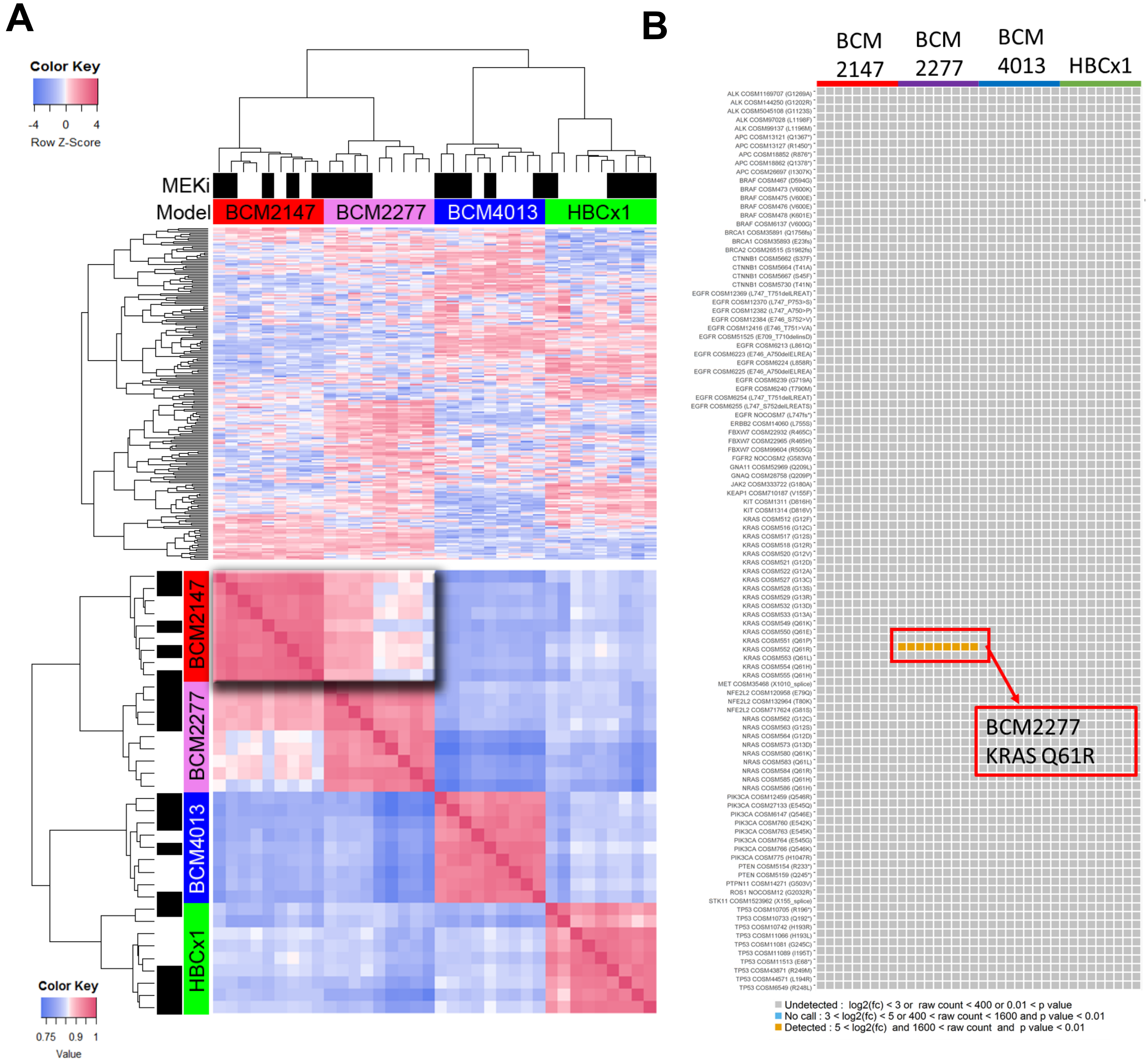

Supplementary Figure 3: BCM-2277 model demonstrates unique gene expression patterns relative to its parental model BCM-2147 and carries a $KRAS^{Q61R}$ *mutation.* A) Heatmap of gene expression patterns for 192 cancer-related mRNAs by model and treatment group. The upper heatmap shows the gene expression patterns while the lower heatmap shows the correlation matrix of similarity across models and treatment groups. The high degree of similarity between BCM-2147 and BCM-2277 treated with trametinib treatment (black) is highlighted compared to vehicle treatment (white). B) Tile plot of mutations detected in PDX models. The only mutations across 104 assayed mutations was a $KRAS^{Q61R}$ mutation detected in 8/8 BCM-2277 samples.

Supplementary Figure 4

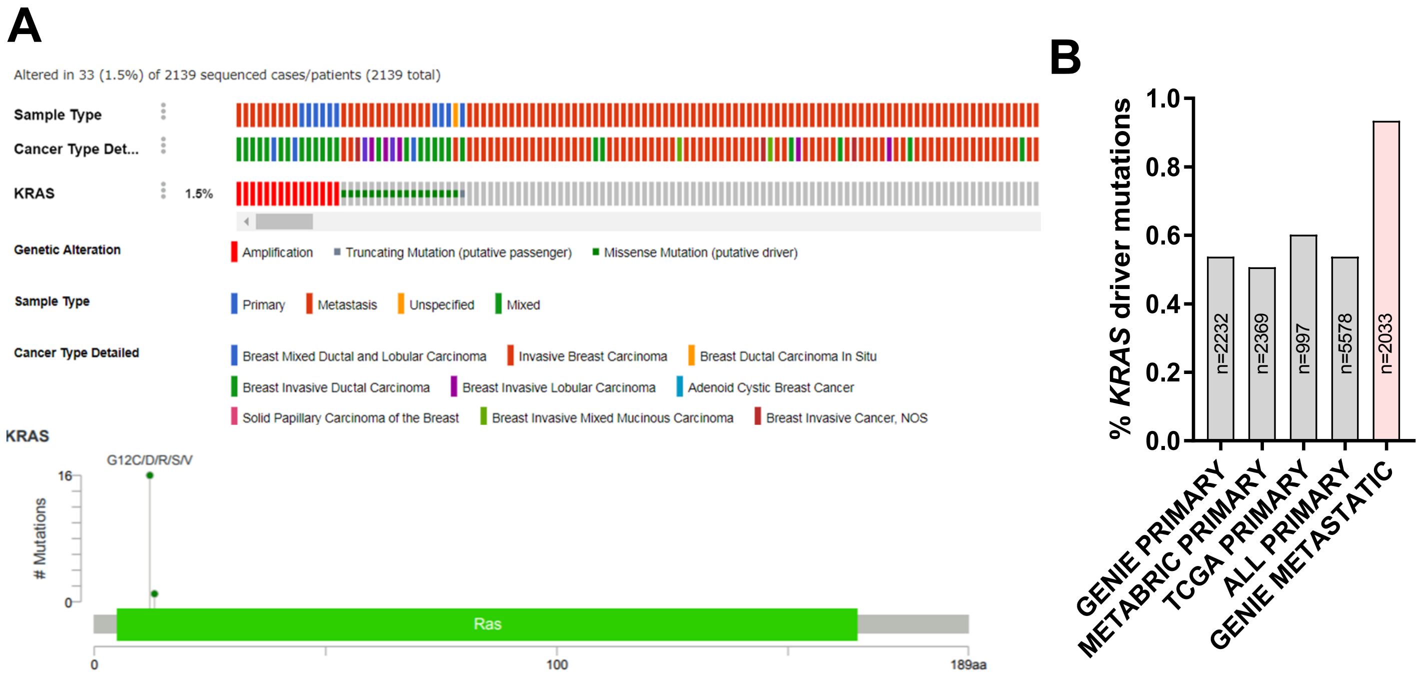

<u>*Supplementary Figure 4: Frequency of KRAS-activating mutations in human breast cancer.*</u> A) Oncoprint analysis of *KRAS* amplifications and mutations in breast cancers in the AACR GENIE data. Activating mutations are enriched in metastatic disease versus primaries. In the lollipop plot (lower), all identified mutations were at G12/G13. B) Enrichment of *KRAS* mutations in metastatic versus primary breast cancer. The frequency of activating (G12/G13 codons) mutations were calculated across each identified database. The GENIE database was stratified by designation as metastatic or primary.

Supplementary Figure 5

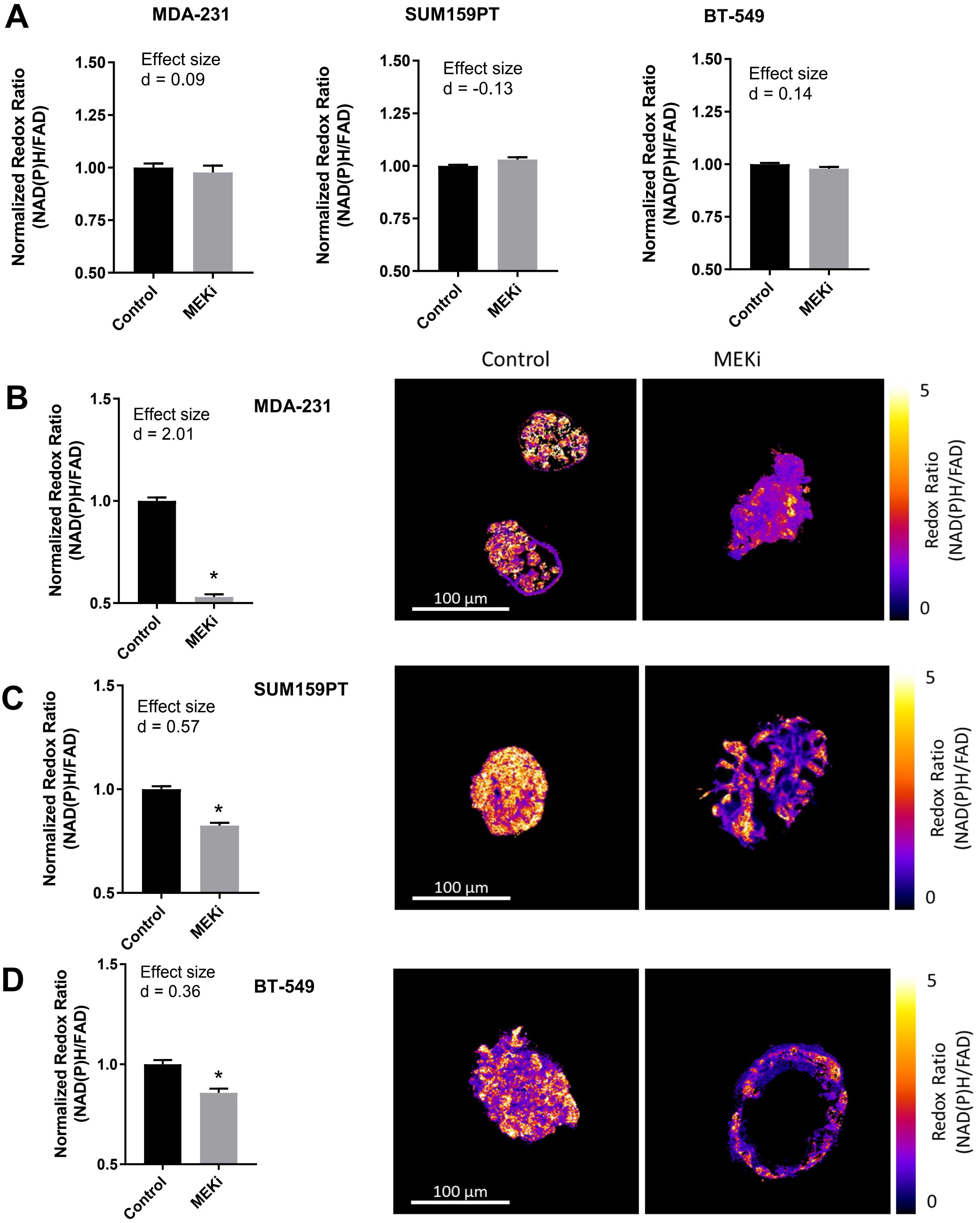

Supplementary Figure 5: Optical metabolic imaging of TNBC cell lines. A) MDA-231, BT549, and SUM159PT cells were grown in 2D and treated for 72hr with 50nM trametinib (MEKi) or DMSO control. After treatment, 50 -500 cells per experiment were imaged (OMI) and redox ratio was calculated as described in methods. Data represent mean ± SEM. B) MDA-231 C) SUM159PT cells and D) BT549 cells were cultured in matrigel as tumor organoids and treated for 72h in 50nM trametinib (MEKi) prior to OMI analysis. At least 10 organoids comprising at least 100 cells were imaged and analyzed in each group. Example organoid images are presented for each cell line/treatment condition. *$p<0.05$.

Supplementary Figure 6

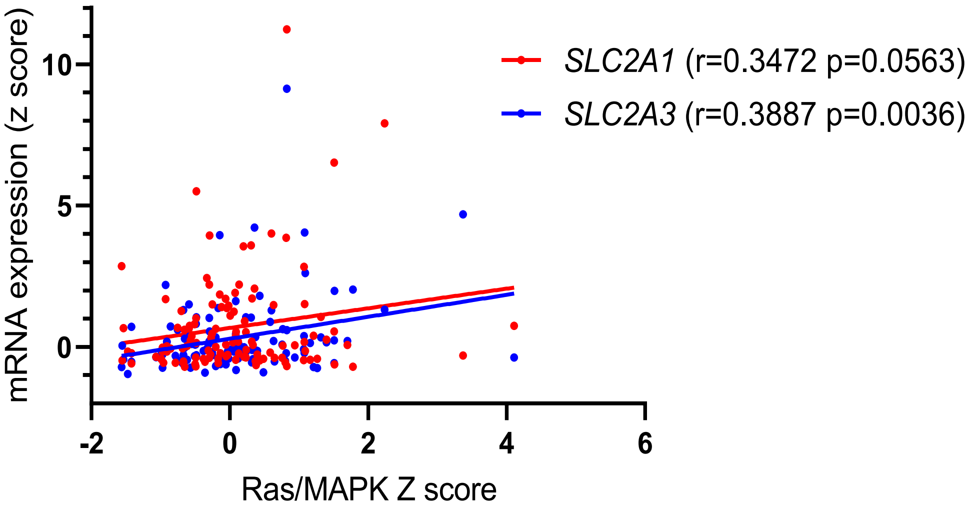

Supplementary Figure 6: Correlation between Ras/MAPK activation and glucose transporters SLC2A1 (GLUT1) and SLC2A3 (GLUT3) in TNBC. mRNA expression Z-scores from 115 breast cancer patients within the TCGA cohort were compared to the previously described Ras/MAPK transcriptional score.

Supplementary Figure 7

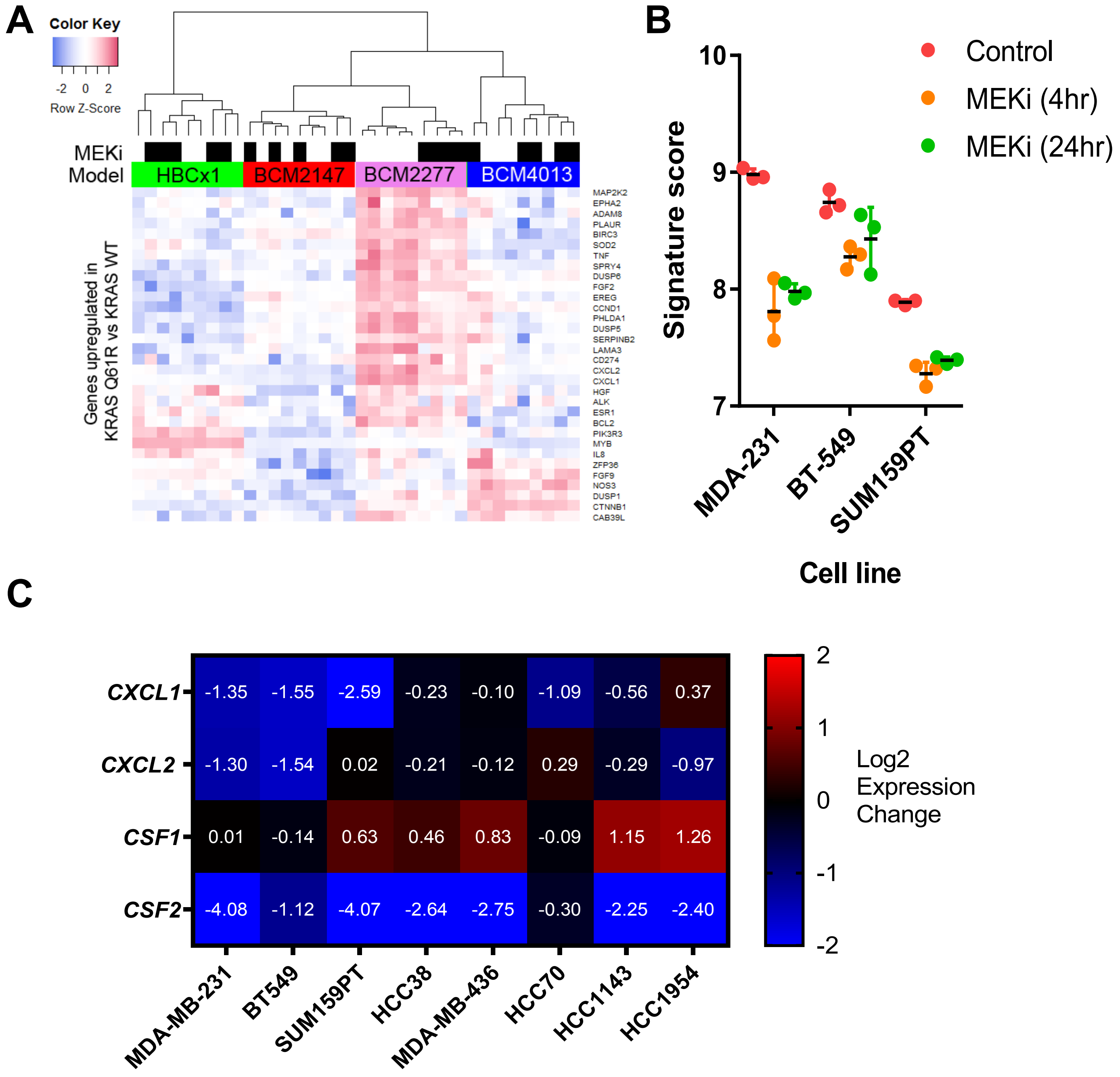

Supplementary Figure 7: Genes upregulated in KRAS^{Q61R} mutant BCM-2277 are repressed by MEK inhibition. A) Heatmap of only mRNAs upregulated in BCM-2277 versus BCM-2147, demonstrating that the majority of these genes are specific to BCM-2277. B) Genes from (A) were used to create a signature score (mean of log2 expression of upregulated genes) and assessed across microarray data from MEKi-treated (4 or 24hr) TNBC cell lines. C) qRTPCR data for *CXCL1, CXCL2, CSF1,* and *CSF2* in a panel of breast cancer cell lines. Expression levels are shown in terms of Log2 values.

Supplementary Figure 8

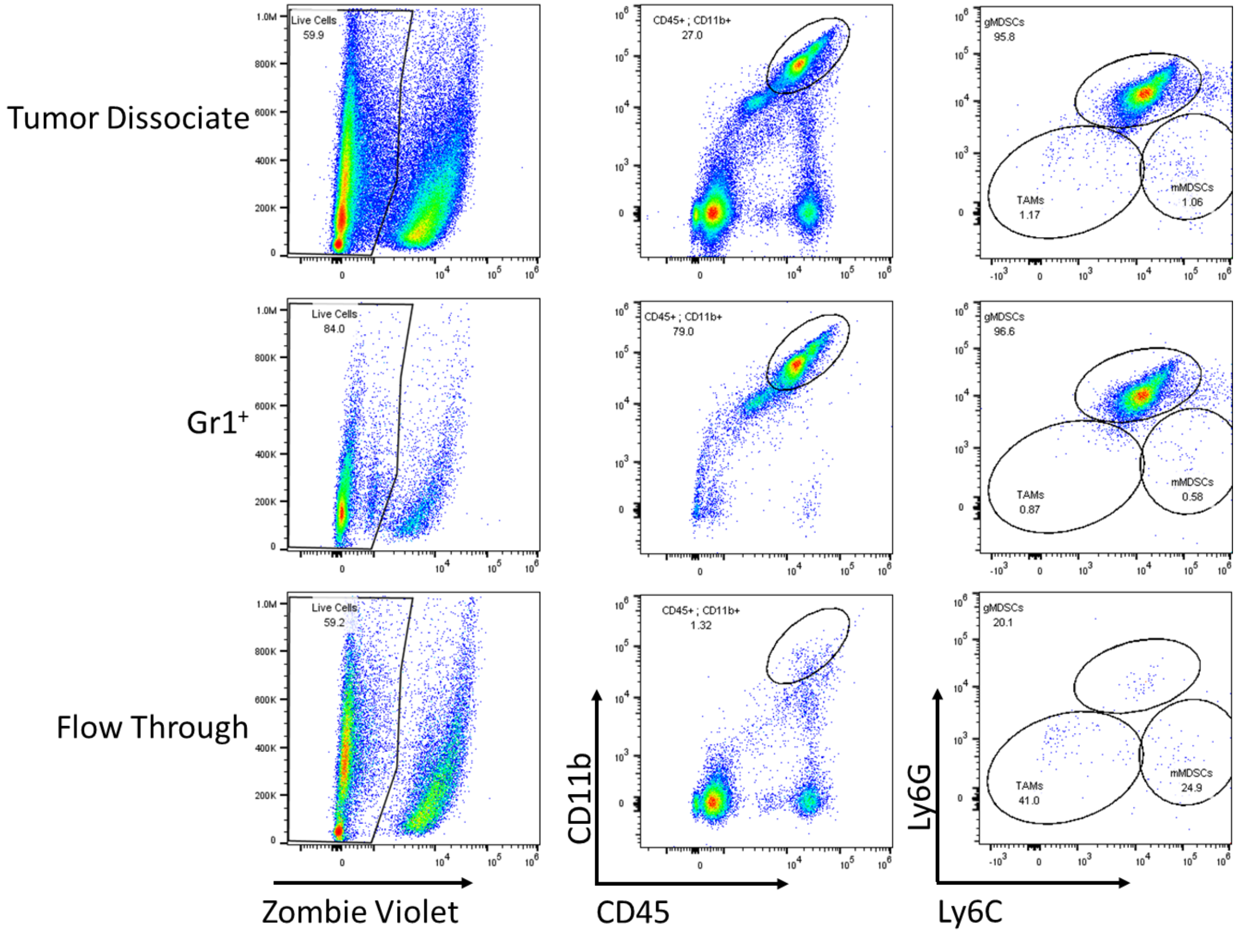

Supplementary Figure 8: Flow cytometry demonstrates column-based enrichment of MDSC populations from BCM-2277 tumors Flow cytometry analysis of tumor dissociates, LY6G^{+} enriched, Gr1^{Dim} enriched, and MDSC-depleted tumor dissociates for CD45/CD11b/Ly6G/Ly6C expression. Cells were analyzed using an Attune Nxt Flow cytometer and FlowJo.

Supplementary Figure 9

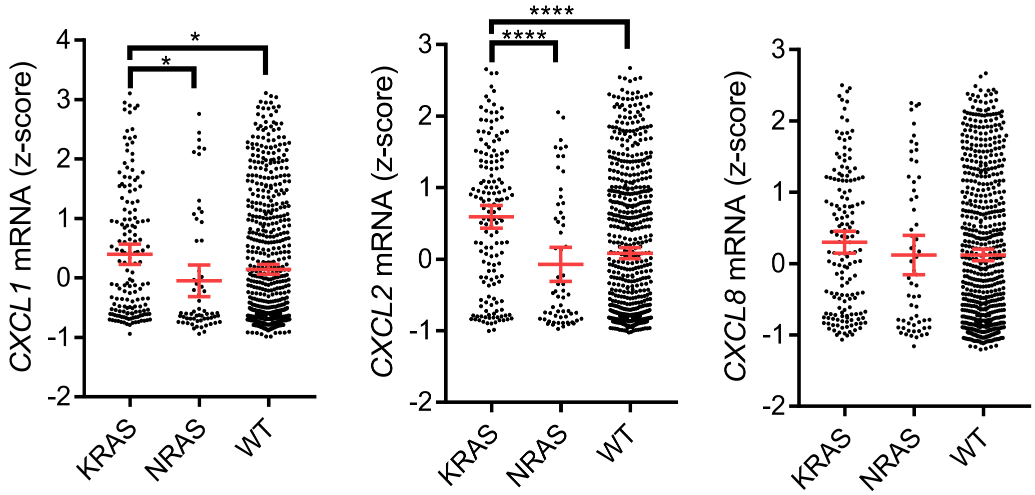

Supplementary Figure 9: *Comparison of CXCL1/2/8 mRNA expression in all cell lines in the Cancer Cell Line Encyclopedia (CCLE), stratified by KRAS or NRAS status.* mRNA expression in all cell lines (multiple tumor types) in the Cancer Cell Line Encyclopedia (CCLE) were stratified by KRAS or NRAS status. *p<0.05 ****p<0.0001 for Tukey's post-hoc analysis (ANOVA p<0.001 for *CXCL1/2*).

Supplementary Figure 10

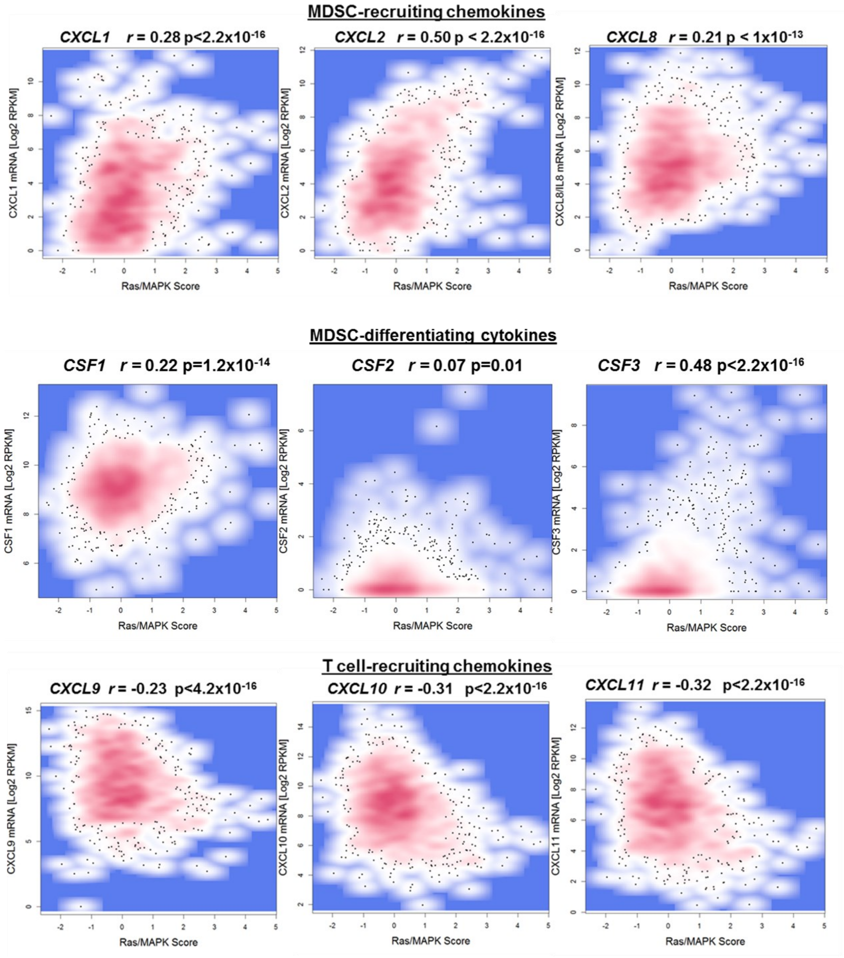

Supplementary Figure 10: Chemokine mRNA correlations with Ras/MAPK activation in breast tumors. mRNA expression Z-scores from >1000 breast cancer patients within the TCGA cohort were compared to the previously described Ras/MAPK transcriptional score. CXCR1/2 (MDSC-recruiting) chemokines are positively associated with Ras/MAPK activation in human breast cancers (TCGA). Colony Stimulating Factor (CSF) family members are positively associated with Ras/MAPK activation in human breast cancers (TCGA). *CXCL9*, *CXCL10*, and *CXCL11* (CXCR3 ligands/T cell-recruiting chemokines) are negatively associated with Ras/MAPK activation in human breast cancers (TCGA).

Supplementary Figure 11

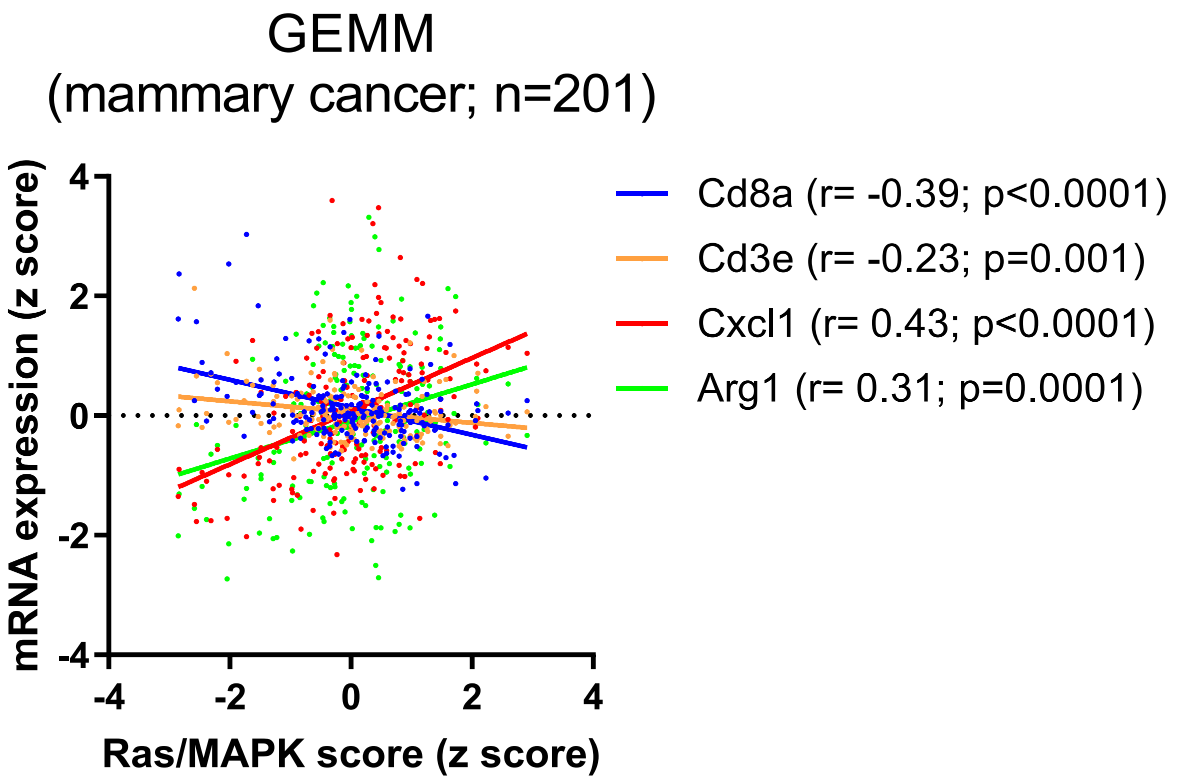

Supplementary Figure 11: Cxcl1 mRNA association with immune infiltrates and Ras/MAPK activation in murine models of breast cancer. Cxcl1, Cd3e, Arg1, and CD8a mRNA levels were assessed from a microarray dataset comprising 201 samples from genetically engineered mouse models of breast cancer(56). Ras/MAPK transcriptional signatures were calculated using the murine homologs for the 50 genes contained in the signature. Graph demonstrates a positive association of Cxcl1 mRNA with Ras/MAPK transcriptional activation
